# Evidence and Potential Mechanism of Action of *Lithospermum erythrorhizon* and Its Active Components for Psoriasis

**DOI:** 10.3389/fphar.2022.781850

**Published:** 2022-05-05

**Authors:** Jiao Wang, Liu Liu, Xiao-Ying Sun, Shuo Zhang, Ya-Qiong Zhou, Kan Ze, Si-Ting Chen, Yi Lu, Xiao-Ce Cai, Jia-Le Chen, Ying Luo, Yi Ru, Bin Li, Xin Li

**Affiliations:** ^1^ Department of Dermatology, Yueyang Hospital of Integrated Traditional Chinese and Western Medicine, Shanghai University of Traditional Chinese Medicine, Shanghai, China; ^2^ Institute of Dermatology, Shanghai Academy of Traditional Chinese Medicine, Shanghai, China; ^3^ Department of Dermatology, Shanghai Skin Disease Hospital, Shanghai, China

**Keywords:** psoriasis, Lithospermum erythrorhizon, Shikonin (SHI), β,β- dimethylacryloyl alkannin (DMA), systematic review, meta-analysis

## Abstract

**Background:** Traditional Chinese medicine is effective in the treatment of psoriasis and can significantly reduce skin inflammation and psoriatic lesions with minimal side effects. Shikonin (SHI) and β,β-dimethylacryloyl alkannin (DMA), the main active components of *Lithospermum erythrorhizon*, have strong anti-inflammatory effects. This systematic review aimed to evaluate the efficacy and safety of *Lithospermum erythrorhizon* and its main active components and to elucidate the potential mechanisms of their action in psoriasis treatment.

**Methods:** PubMed, Embase, Cochrane Central Register of Controlled Trials, China National Knowledge Infrastructure, Chinese Scientific Journals, Wan Fang, and Chinese Biomedicine databases were systematically searched for articles published between 1 January 1970, and 31 February 2021. We included clinical and preclinical studies that examined the effects of *Lithospermum erythrorhizon* and its active components on psoriasis. All data were analyzed using RevMan 5.3 software. The Cochrane and SYRCLE’s risk-of-bias tools were used to assess the quality of all studies.

**Results:** Eleven clinical trials including 1024 participants and 23 preclinical studies were assessed. Meta-analysis showed that when treating patients with psoriasis, the Chinese herbal medicine (CHM) formulas with *Lithospermum erythrorhizon* as the sovereign herb can significantly improve psoriatic dermatitis, which can significantly reduce the psoriasis area and severity index (PASI) score (mean difference [MD] = -2.00, 95% confidence interval [CI] [-3.19, -0.80], *p* = 0.001; I^2^ = 85%). The incidence rates of diarrhea (risk ratio = 0.21, 95% CI [0.06, 0.81], *p* = 0.02) were higher in the CHM formulas group than in the control group, whereas other adverse events were not significantly different between the two groups (*p* > 0.05). We evaluated the PASI score of mice on day 7 and found that SHI and DMA also alleviated psoriatic lesions (MD = -3.36, 95% CI [-4.67, -2.05], *p* < 0.00001, I^2^ = 94%). Furthermore, the epidermal thickness decreased more after SHI or DMA treatment than in the control group (MD = -34.42, 95%CI [-41.25, -27.59], *p* < 0.00001, I^2^ = 93%). Based on preclinical studies, we also summarized and mapped the mechanisms of SHI and DMA in the treatment of psoriasis.

**Conclusion:** Available findings demonstrated that *Lithospermum erythrorhizon* combined with other conventional treatments is useful in treating psoriasis. Preclinical evidence has shown that the active components of *Lithospermum erythrorhizon* exhibit a potential anti-inflammatory effect, promote keratinocyte apoptosis, inhibit keratinocyte proliferation and angiogenesis, and block the cell cycle. In summary, our findings suggest that *Lithospermum erythrorhizon* and its active components can be used to treat psoriasis.

## 1 Introduction

Psoriasis is a chronic inflammatory skin disease characterized by excessive proliferation of epidermal keratinocytes and hyperkeratosis caused by infiltration of dermal inflammation (Hawkes et al., 2018). The typical clinical manifestations of psoriasis are scaly erythema, thick scaly plaques, membranous phenomena, and punctate hemorrhage (Li et al., 2016). Approximately 125 million people worldwide suffer from psoriasis (Armstrong and Read, 2020). A survey in Israel found that the prevalence of psoriasis increased from 2.5% in 2011 to 3.8% in 2017 (Schonmann et al., 2019). Studies have shown that psoriasis is significantly associated with hyperuricemia, chronic obstructive pulmonary disease, dementia, and other diseases (Li et al., 2015; Li et al., 2016; Chen et al., 2020). This severely affects the quality of life of patients and imposes a substantial burden on the global economy.

The pathogenesis of psoriasis remains unclear; however, studies have shown that the interleukin (IL)-23/IL-17 immune pathway plays a vital role in promoting its development and progression. IL-17 is a key effector cytokine in this pathway (Fan et al., 2015; Blauvelt and Chiricozzi, 2018). The expression of IL-17 is significantly upregulated in the skin and bloodstream of psoriasis patients, and IL-17-produced T-cells that are also highly expressed (Chiricozzi et al., 2016). Currently, the following drugs are mainly used to treat psoriasis in clinical practice: retinoids, vitamin D, biologics, and cyclosporin A, the phosphodiesterase 4 inhibitor (Ogawa et al., 2018). However, these treatments have certain toxicities, multiple side effects, and involve high economic costs. Therefore, an effective alternative treatment for psoriasis is required. The treatment of psoriasis using traditional Chinese medicine (TCM) is favored by the majority of patients because of its remarkable curative effects and minimal side effects (Luo et al., 2020). TCM believes that the core pathogenesis of psoriasis is “blood based, blood heat first, and blood stasis throughout the whole process of the disease,” and proposes a method of cooling blood and latent Yang and promoting blood circulation and dissipating blood stasis as the key treatment methods for the management of psoriasis (Li, 2019).

**FIGURE 1:**
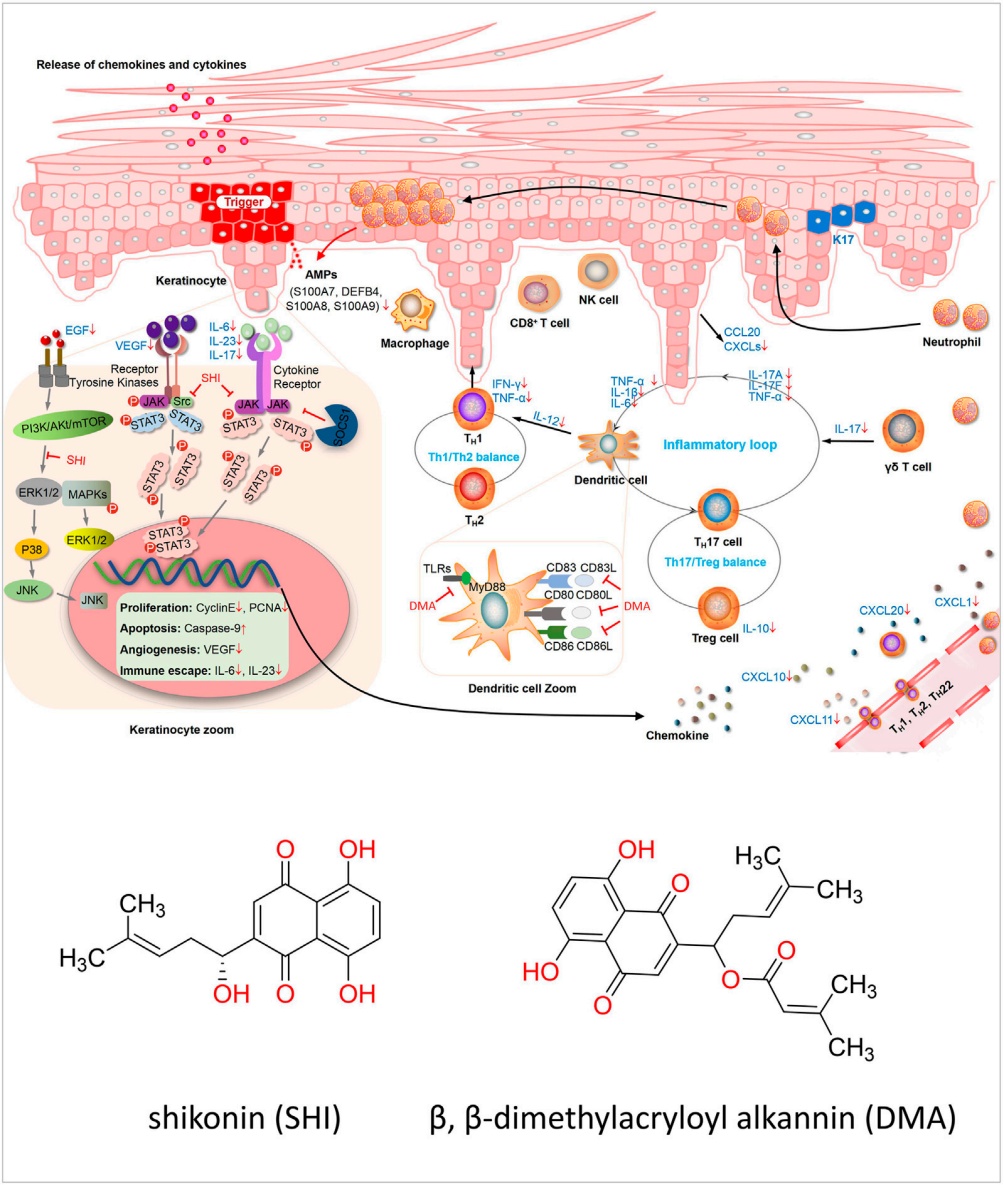
Diagram of the mechanism of preclinical *in vitro.* The pathogenesis of psoriasis is related to the KC/DC/T loop when treated with SHI and DMA, and the cells in this loop are all changed. In HaCaT cells, the cell cycle caused a decrease in cell proliferation and an increase in apoptosis. Furthermore, intracellular signaling pathways were inhibited, resulting in downstream transcription factors such as Gab1 and Gab2. SHI and DMA affected the activity of DCs, and the secretion of inflammatory factors, such as IL-23, was also reduced. For T-cells, Th1, Th2 and TH17 cells were all affected. SHI and DMA balanced Th1 and Th2 cells and inhibited IL-17 levels.

*Lithospermum erythrorhizon*, Zicao, *Lithospermum erythrorhizon Siebold and Zucc*, Puccoon, and Gromwell, a perennial herbal plant of the family Boraginaceae, are commonly used in Chinese medicine and were first recorded in Shennong BencaoJing (Shennong’s Classic of Materia Medica), the earliest complete Pharmacopoeia of China. It is the dry root of Arnebia euchroma (Royle) Johnst or Arnebia guttata Bunge. In addition, the root bark of the same family of Onosma paniculata Bur.et Franch., Onosma confertum W. W. Smith, Onosma exsertum Hemsl, and Onosma hookeri clarke Var.Longiflorum Duthie are also used as medicines. The active components of *Lithospermum erythrorhizon* are mainly divided into two categories: fat-soluble components and naphthoquinone compounds (Lu et al., 1998). At present, 28 naphthoquinone compounds have been isolated from Lithospermum erythrorhizo, mainly shikonin (SHI) and β,β-dimethylacryloyl alkannin (DMA), and their chemical structures are shown in Figure 1. The other is water-soluble components, mainly a mixture of polysaccharides and glycoproteins. Polysaccharides have good immunomodulatory activity, although little research has been conducted to date (Yi, 2005). Previous studies have reported that a high-performance liquid chromatographic method using diode-array detection was used to quantitatively analyze the active components in the TCM *Lithospermum erythrorhizon*, including SHI and DMA (Hu et al., 2006). Modern pharmacological studies have shown that these components, especially SHI, have a wide range of biological activities, including anti-inflammatory, antiviral, antitumor, anti-estrogen, and immunomodulatory activities (Lin et al., 2013). Furthermore, recent studies have shown that SHI had strong anti-inflammatory effects in psoriasis models.

Nevertheless, there has been no systematic review of the therapeutic efficacy and safety of *Lithospermum erythrorhizon* for psoriasis, and the mechanism of SHI has not been systematically evaluated and summarized. Therefore, we conducted a systematic review and meta-analysis to review the recent literature on *Lithospermum erythrorhizon* and its active components in the treatment of psoriasis and to gather current preclinical and clinical evidence regarding its efficacy and mechanism of action.

## 2 Methods and Analysis

### 2.1 Search strategy

The following databases were searched from 1 January 1970 (the inception), to 31 February 2021, for the literature on the use of *Lithospermum erythrorhizon* and its components in the treatment of psoriasis: PubMed, Embase, Cochrane Central Register of Controlled Trials, Web of Science, Chinese Biomedical Literature Database, China National Knowledge Infrastructure, WanFang Database for Chinese Technical Periodicals, and VIP Database. We combined medical subject headings and free text words to retrieve all 36 relevant studies. The following keywords were used: Psoriasis, Psoriasis Pustulosis of Palms and Soles, Pustulosis Palmaris et Plantaris, Palmoplantaris Pustulosis, and Pustular Psoriasis of Palms and Soles. *Lithospermum erythrorhizon*s, Puccoon, Gromwell, Gromwells, Lithospermi, Zicao, LPM, shikonin, alkannin, acetylshikonin, deoxyshikonin, Isobutyshi-konin, β,β-dimethylacrylshikonin, β,hydroxyisovalerylshikonin, alkannan, and α-methyl-n-butyrylshikonin.

### 2.2 Inclusion Criteria

The inclusion criteria for clinical studies were as follows: 1) randomized clinical trials (RCTs); 2) human studies only; 3) patients with definite diagnostic standards of psoriasis, regardless of age, sex, or ethnicity, as the study population; and 4) as the intervention, the Chinese herbal medicine (CHM) formulas (Cheng et al., 2017) with *Lithospermum erythrorhizon* as the sovereign herb. “Sovereign herb” is a Chinese medicine term used to describe the medicine in the prescription that has the strongest therapeutic effect on the main disease or syndrome (Hu, 2016), and 5) standardized therapeutic evaluation (total effectiveness) as the outcome.

For preclinical studies, we selected studies that satisfied the following inclusion criteria: 1) experimental animals, including rats and mice; 2) intervention drugs, SHI, and DMA; and 3) the effects of SHI or DMA on an animal model of psoriasis. Studies that met the following criteria were excluded: 1) combination with other drugs; 2) non-animal research; 3) no pre-determined outcome index; 4) double publications; and 5) case reports, clinical experience, conferences, and scientific and technological achievements.

### 2.3 Data Extraction

Two authors (JW and LL) independently screened the literature and extracted the data. Titles and abstracts were read for the preliminary screening, and the full texts were read to determine the studies eligible for inclusion. The following information was extracted from clinical studies: first author, publication year, sample size, age, sex, duration of psoriasis, characteristics of the intervention and control groups, outcome, and information on risk of bias assessment, efficacy, and adverse events. The following information from preclinical studies was extracted: first author’s name; year of publication; characteristics of the animals, including species, sex, and weight; model establishment and anesthesia methods; intervention characteristics, including dose and route of administration; and result indicators.

### 2.4 Risk-Of-Bias Assessment

The risk of bias was independently assessed by four researchers (SZ, X-CC, KZ, and J-LC). For clinical research, we used the risk-of-bias tool for RCTs recommended by the Cochrane Handbook for Systematic Reviews of Interventions, and the systematic review centre for laboratory animal experimentation (SYRCLE) animal research risk-of-bias tool provided on the website for preclinical research. The evaluation parameters of the risk-of-bias tool for RCTs were as follows: random sequence generation, allocation concealment, blinding of participants and personnel, blinding of the outcome assessment, incomplete outcome data, selective reporting, and other biases. The SYRCLE tool covers six aspects of bias: selection, performance, detection, attrition, reporting, among others. Each term was divided into three grades: a judgment of “Yes” implying low risk, a judgment of “No” implying high risk, and a judgment of “Unclear” implying insufficient details to assess the risk of bias. The four reviewers worked together to evaluate the quality of the clinical and preclinical studies. Disagreements between the two assessors during the evaluation process were resolved through mutual consultation. If differences persisted, a third researcher (JW) participated in the discussion to reach a consensus.

### 2.5 Statistical Analysis

RevMan 5.3 software, provided by the Cochrane Collaboration (London, England), was used for the data analyses. Risk ratios (RRs) with 95% confidence intervals (CIs) were calculated for dichotomous data, whereas mean differences (MDs), and standard MDs with 95% CIs were calculated for continuous data. Heterogeneity was assessed using the chi-square test and the I^2^ statistic to identify whether the results of different studies were homogenous. An I^2^statistic of 0–50% was interpreted as unimportant, and a fixed effects model was applied; however, for I^2^values > 50%, which likely indicates moderate to considerable heterogeneity, a random-effects model was used.

## 3 Results

### 3.1 Selection and Characteristics of Studies

#### 3.1.1 Clinical Studies

From the 683 initially retrieved studies, 464 duplicate and irrelevant articles were removed, and 167 case reports, preclinical trials, and reviews were excluded after abstract and full-text reviews. There were 15 articles in which *Lithospermum erythrorhizon* was not the sovereign herb, 7 articles were not included in the criteria, 10 articles were not RCTs, 4 articles had incomplete basic information, 1 article did not have efficacy criteria, 1 article was published repeatedly, and 3 were other types of articles (such as conference papers and letters). These 41 articles were excluded from the analyses. Finally, 11 RCTs (Han, 2006; Shi et al., 2008; Li, 2013; Ma, 2013; Sun, 2016; Chen and Lei, 2018; Luo et al., 2018; Zhang, 2018; Su, 2019; Gao et al., 2020; Zhang, 2020) met the inclusion criteria and were included in our systematic review (Supplementary Figure S1). All included studies were performed in China between 1970 and 2021.

#### 3.1.2 Preclinical Studies

We searched 683 relevant articles, and 462 were retained after deleting duplicates and irrelevant articles; 171 were excluded after reading the titles and abstracts. The main reasons for exclusion were as follows: 1) case reports, 2) clinical trials, and 3) review articles. Then, by reading the full text, 27 articles were excluded for at least the following reasons: 1) combined with other medicines, 2) no predetermined outcome index, 3) double publication, and 4) others (such as conference papers, letters, etc.). Finally, we identified 23 articles that met these requirements. Among them, 2 articles contained both animal and cell experiments (Supplementary Figure S1).

### 3.2 Characteristics of Included Studies

#### 3.2.1 Clinical Studies

Twelve trials involved 1,024 participants in the analysis, including 565 in the experimental group and 459 in the control group. This systematic review included six interventions, including decoctions containing *Lithospermum erythrorhizon* (LD) and CHM formulas with *Lithospermum erythrorhizon* as the sovereign herb, including Zilian, Liangxue Jiedu, Mahuang Zimei, Zicao Huoxue, Zicao, and Zicao Biejia Siwu decoctions. Topical CHM formulas containing *Lithospermum erythrorhizon* (LT), ointment, oil, or lotion with *Lithospermum erythrorhizon* as the main herb, including Zicao ointment, Zicao oil, and Zicao Quyin lotion; other oral drugs (OOD), including Diyin tablets, compound aminopeptin tablets, Liangxue Huoxue decotion, TCM, acitretin, and Tripterygium wilfordii tablets; and other topical therapies (OTT), including Vaseline, Pulian ointment, Capitriol ointment, TCM steam therapy, Narrow Bound Ultra Violet B Light (NB-UVB), and tacrolimus ointment. According to the 2017 CONSORT Extension for Chinese Herbal Medicine Formulas (Cheng et al., 2017), we have made a checklist of CHM formulas for clinical research. The checklist includes the following items: composition and dosage of CHM formulas (Chinese Pinyin name and Latin name), authentication method, principles, rationale, and interpretation of forming the CHM formulas, reference(s) as to the effificacy, pharmacologic study results, production method, quality control of each ingredient and of the product, safety assessment, dosage, administration route, and preparation method of the CHM formulas (Supplementary Table S1). Two studies (Han, 2006; Luo et al., 2018) compared the combined use of LD and ODD with that of OOD alone. Three studies (Shi et al., 2008; Li, 2013; Zhang, 2020) compared the efficacy of LD and OOD. Two studies (Ma, 2013; Sun, 2016) compared the efficacy of LT and OOD in combination with the efficacy of OOD alone. A previous study (Chen and Lei, 2018) compared the efficacy of LT and OTT. Two studies (Zhang, 2018) (Gao et al., 2020) compared the efficacy of LD combined with OTT and that of OTT alone. A previous study (Su, 2019) compared the efficacy of LT combined with OTT and that of OTT alone. One study (Zhang, 2018) divided the experimental group of *Lithospermum erythrorhizon* into high and low doses. Six studies reported adverse events (Han, 2006; Shi et al., 2008; Ma, 2013; Chen and Lei, 2018; Luo et al., 2018; Zhang, 2018). Recurrence was reported in one study (Han, 2006). To evaluate the efficacy, we combined healing, significantly effective, and effective outcomes into one positive category, while invalid was considered a negative category. These data were extracted as dichotomous outcomes. Six studies (Shi et al., 2008; Li, 2013; Sun, 2016; Chen and Lei, 2018; Zhang, 2018; Su, 2019) reported the psoriasis area and severity index (PASI) score, eleven (Han, 2006; Shi et al., 2008; Li, 2013; Ma, 2013; Sun, 2016; Chen and Lei, 2018; Luo et al., 2018; Zhang, 2018; Su, 2019; Gao et al., 2020; Zhang, 2020) reported an effective rate, one (Su, 2019) reported a visual analog scale score, two (Su, 2019) (Gao et al., 2020) reported skin barrier function, three (Luo et al., 2018; Zhang, 2018; Su, 2019) reported serum measures, and one (Luo et al., 2018) reported quality of life (Supplementary Table S2).

#### 3.2.2 Preclinical Studies

Preclinical research mainly includes animal and cell experiments. For animal experiments, one study (Zhao, 2016a) was a doctoral thesis and the other five (Yu et al., 2019; Wang et al., 2016; Wang et al., 2015; Lan et al., 2020; Zhang et al., 2019) were published in English. All six studies used BALB/c mice. The body weight of the mice varied between 17 and 25 g, and two of the studies (Zhao, 2016a; Yu et al., 2019) did not mention the weight of the mice. One study (Zhao, 2016a) used 45 female mice, whereas five others (Yu et al., 2019; Wang et al., 2016; Wang et al., 2015; Lan et al., 2020; Zhang et al., 2019) used 140 male mice. All mice were imiquimod-induced psoriasis models. To induce anesthesia, two studies (Wang et al., 2016; Wang et al., 2015) used sodium pentobarbital, while the remaining four did not mention anesthesia. The methods of intervention mainly included the following: three studies (Zhao, 2016a; Yu et al., 2019; Zhang et al., 2019) used the SHI intervention, two (Wang et al., 2016; Wang et al., 2015) used the DMA intervention, and one (Lan et al., 2020) used the SHI oil (SO) intervention. Among the outcome measures, five studies (Zhao, 2016a; Yu et al., 2019; Wang et al., 2015; Lan et al., 2020; Zhang et al., 2019) evaluated the PASI score, two (Zhao, 2016a; Zhang et al., 2019) evaluated hematoxylin and eosin (HE) staining and IL-17A, and three (Zhao, 2016a; Yu et al., 2019; Wang et al., 2015) evaluated epidermal thickness. All SHIs or DMAs used in the animal experiments were purchased from a company, and only one study (Zhang et al., 2019) did not mention the source. Only two studies (Lan et al., 2020; Zhang et al., 2019) mentioned SHI concentrations of 20 mg/ml and 0.5766 mg/ml. Two studies (Wang et al., 2016; Lan et al., 2020) did not describe the specific doses of SHI or DMA; other studies reported doses of 2.5, 5, and 10 mg/kg/day; 5 and 10 mg/kg/day; and 6.25, 12.5, and 25 mg/kg/day. The routes of administration were intraperitoneal injection in one study (Zhao, 2016a; Wang et al., 2016), topical injection in one study (Lan et al., 2020), and intragastric administration in all others. The detailed characteristics of the included studies are summarized in Supplementary Table S3. The main active components of *Lithospermum erythrorhizon* in the treatment of psoriasis were identified using *in vitro* experiments in 17 studies involving 5 cell types, namely HaCaT (Xing, 2010; Wang et al., 2011; Zhu, 2013; Xu et al., 2014; Xie, 2015; Zhao, 2016b; Liu, 2017; Yu et al., 2019; Lan et al., 2020), dendritic cells (DCs) (Wang, 2014; Wang et al., 2015), Jurkat E6-1 (Liu, 2018), peripheral blood mononuclear cells (PBMCs) (Qu, 2010; Zhang, 2011; Wang, 2017), and Colo-16 (Wu and Zhou, 2003; Sun et al., 2004) (Supplementary Table S4). We created a checklist of SHI and DMA *in vivo* and *in vitro* studies that includes access, route of administration, experimental concentration, dose, and preparation method (Supplementary Table S5).

### 3.3 Risk of Bias

#### 3.3.1 Clinical Studies

Most of the included trials had low methodological quality, and Supplementary Figure S2 shows the risk of bias in the 11 included studies. Six experiments (Han, 2006; Ma, 2013; Sun, 2016; Chen and Lei, 2018; Su, 2019; Gao et al., 2020) used the random number table method, one study (Zhang, 2018) used the random envelope method, and four (Shi et al., 2008; Li, 2013; Luo et al., 2018; Zhang, 2020) introduced the random method only. Allocation concealment was not mentioned in any of the articles. Furthermore, none of the studies recorded hidden or blind allocation of participants, key personnel, or outcome assessments, and all studies reported complete results (Supplementary Figure S2, S3).

#### 3.3.2 Preclinical Studies

None of the six studies (Wang et al., 2015; Zhao, 2016a; Wang et al., 2016; Yu et al., 2019; Zhang et al., 2019; Lan et al., 2020) mentioned whether the method was used to generate the allocation sequence, all possible prognostic factors or animal characteristics, the method of hiding the allocation sequence, the blinding of the trial nurses and researchers, the random method used, or whether the evaluator used the blind method for conducting animal selection and outcome evaluation. However, all six studies reported random housing, incomplete outcome data, selective outcome reporting, and other sources of bias. Therefore, the overall quality of the included studies was relatively low (Supplementary Figure S4).

### 3.4 Primary Outcome of Clinical Studies

#### 3.4.1 PASI

The PASI score is a score of the degree of psoriasis lesions, which is a quantitative indicator of the severity of psoriasis. The significance is to reflects the condition of psoriasis with specific numbers and can be used as a scientific basis for judging the treatment effect. In our included studies, only six articles (Shi et al., 2008; Li, 2013; Sun, 2016; Chen and Lei, 2018; Zhang, 2018; Su, 2019) reported the PASI score. We evaluated the PASI scores of all intervention and control groups. The total PASI score showed that CHM formulas with *Lithospermum erythrorhizon* as the sovereign herb significantly improved psoriatic dermatitis (MD = −2.00, 95% CI −3.19, −0.80], *p* = 0.001; I^2^ = 85%). We also conducted subgroup analyses based on the results of this study. Specifically, comparing LD and OOD (MD = −1.14, 95% CI [−3.32, −1.05], *p* = 0.31, I^2^ = 82%), and comparing LT and OTT (MD = −0.40, 95% CI [−1.30, 0.50], *p* = 0.38); the curative effect was similar, and there was no statistical difference. Moreover, the combination of LD and OTT was better than that of OTT alone (MD = −3.04, 95% CI [−5.00, −1.08], *p* = 0.002, I^2^ = 63%), the combination of LT and OOD was better than that of OOD alone (MD = −1.50, 95% CI [−2.51, −0.49], *p* = 0.004). The combination of LT and OTT was better than that of OTT alone (MD = −4.11, 95% CI [−5.36, −2.86], *p* < 0.00001), and the difference was statistically significant (Table 1).

**TABLE 1:** Subgroup analysis of PASI in clinical studies.

References	Comprison	Change from Baseline (Mean ± SD)		Mean Difference [95%CI]	*p* value
		Experiment	Control		
**1.PASI**					
**1.1 LD + OTT VS. OOD + OTT**					
Li et al., 2013	LD + OTT VS. OOD + OTT	3.15 ± 3.67	5.46 ± 4.63	−2.31 [−3.80, −0.82]	—
Shi et al., 2008	LD + OTT VS. OOD + OTT	4.39 ± 2.77	4.47 ± 2.97	−0.08 [−1.19, 1.03]	—
Subtotal (95% CI) I^2^ = 82%				−1.14 [−3.32, 1.05]	*p* = 0.31
**1.2 LD + OTT VS. OTT**					
Zhang, et al., 2018(a)	LD + OTT VS. OTT	6.89 ± 2.62	10.89 ± 2.53	−4.00 [−5.59, −2.41]	—
Zhang, et al., 2018(b)	LD + OTT VS. OTT	8.89 ± 3.41	10.89 ± 2.53	−2.00 [−3.77, −0.23]	—
Subtotal (95% CI) I^2^ = 63%				−3.04 [−5.00, −1.08]	*p* = 0.002
**1.3 LT + OOD VS. OOD**					
Sun, et al., 2016	LT + OOD VS. OOD	15.10 ± 1.50	16.60 ± 2.30	−1.50 [−2.51, −0.49]	—
Subtotal (95% CI)				−1.50 [−2.51, −0.49]	*p* = 0.004
**1.4 LT + OTT VS. OTT**					
Su, et al., 2019	LT + OTT VS. OTT	4.77 ± 3.12	8.88 ± 3.26	−4.11 [−5.36, −2.86]	—
Subtotal (95% CI)				−4.11 [−5.36, −2.86]	*p* < 0.00001
**1.5 LT VS. OTT**					
Chen, et al., 2018	LT VS. OTT	2.80 ± 1.20	3.20 ± 2.20	−0.40 [−1.30, 0.50]	—
Subtotal (95% CI)				−0.40 [−1.30, 0.50]	*p* = 0.38
Total (95% CI) Random-effects I^2^ = 86%				−1.57 [−2.95, −0.20]	*p* = 0.03

### 3.5 Secondary Outcomes of Clinical Studies

#### 3.5.1 Efficacy

All articles (Han, 2006; Shi et al., 2008; Li, 2013; Ma, 2013; Sun, 2016; Chen and Lei, 2018; Luo et al., 2018; Zhang, 2018; Su, 2019; Gao et al., 2020; Zhang, 2020) reported the effective rate, and we analyzed it as one of the secondary outcomes. Overall, compared with other drugs, CHM formulas with *Lithospermum erythrorhizon* as the sovereign herb significantly improved the condition of patients with psoriasis (RR = 1.19, 95% CI [1.12, 1.26], *p* < 0.00001, I^2^ = 4%). We divided the patients into six subgroups, and the results showed a statistical difference between LD + OTT and OOD + OTT (RR = 1.23, 95% CI [1.03, 1.48], *p* = 0.02, I^2^ = 68%), LD + OOD and OOD (RR = 1.15, 95% CI [1.01, 1.30], *p* = 0.03, I^2^ = 0%), LT + OTT and OTT (RR = 1.17, 95% CI [1.03, 1.33], *p* = 0.02), indicating that in terms of the curative effect of CHM formulas with *Lithospermum erythrorhizon* as the sovereign herb in the treatment of psoriasis, the curative effect of LD was better than that of OOD alone, the effects of LT and OTT were better than those of OOD alone, and the effects of LT and OTT were better than those of OTT alone. There was no statistical difference between LD + OTT and OTT (*p* = 0.10), LT + OOD and OOD (*p* = 0.06), and LT and OTT (*p* = 0.54); namely, the effects of LD and OTT were not significantly different from those of OTT alone. The effects of LD and OOD were similar to that of OOD alone. LT and OTT were similar (Table 2).

**TABLE 2:** Subgroup analysis of effective rate in clinical studies.

References	Comparison	Experimental Group		Control Group		Risk Ratio [95%CI]	*p* value
		No. of Events	Total	No. of Events	Total		
**2.Effective rate**							
**2.1 LD + OTT VS. OOD + OTT**							
Li, et al., 2013	LD + OTT VS. OOD + OTT	56	60	38	60	1.47 [1.20, 1.81]	—
Shi, et al., 2008	LD + OTT VS. OOD + OTT	93	105	30	36	1.06 [0.90, 1.25]	—
Zhang 2020	LD VS. OOD	—	—	—	—	1.23 [1.04, 1.45]	—
Subtotal (95% CI) I^2^ = 68%						1.23 [1.03, 1.48]	*p* = 0.02
**2.2 LD + OTT VS. OTT**							
Zhang, et al., 2018	LD + OTT VS. OTT	41	60	13	30	1.19 [1.02, 1.40]	—
Gao, et al., 2020	LD + OTT VS. OTT	43	45	36	45	1.58 [1.01, 2.46]	—
Subtotal (95% CI) I^2^ = 53%						1.30 [0.95, 1.79]	*p* = 0.10
**2.3 LD + OOD VS. OOD**							
Han 2006	LD + OOD VS. OOD	47	80	20	40	1.18 [0.82, 1.68]	—
Luo, et al., 2018	LD + OOD VS. OOD	48	50	42	50	1.14 [1.00, 1.31]	—
Subtotal (95% CI) I^2^ = 0%						1.15 [1.01, 1.30]	*p* = 0.03
**2.4 LT + OOD VS. OOD**							
Ma 2013	LT + OOD VS. OOD	42	44	32	39	1.16 [0.99, 1.37]	—
Sun, et al., 2016	LT + OOD VS. OOD	36	58	13	30	1.43 [0.91, 2.26]	—
Subtotal (95% CI) I^2^ = 14%						1.20 [0.99, 1.46]	*p* = 0.06
**2.5 LT + OTT VS. OTT**							
Su, et al., 2019	LT + OTT VS. OTT	49	50	42	50	1.17 [1.03, 1.33]	—
Subtotal (95% CI)						1.17 [1.03, 1.33]	*p* = 0.02
**2.6 LT VS. OTT**							
Chen, et al., 2018	LT VS. OTT	24	30	22	30	1.09 [0.82, 1.44]	—
Subtotal (95% CI)						1.09 [0.82, 1.44]	*p* = 0.54
Total (95% CI) Random-effects I^2^ = 4%						1.19 [1.11, 1.27]	*p* < 0.0001

#### 3.5.2 Adverse Events

Seven trials evaluated the side effects of CHM formulas with *Lithospermum erythrorhizon* as the sovereign herb in patients with psoriasis. The most common adverse reaction was diarrhea. Two studies (Zhang, 2018) (Zhang, 2020) have shown that the combined use of LD and OTT can increase the risk of diarrhea more than that of OTT alone, and the difference was also statistically significant (RR = 0.21, 95% CI [0.06, 0.81], *p* = 0.02, I^2^ = 0%). Concurrently, two studies (Han, 2006; Luo et al., 2018) have shown that the combined use of LD and OOD cannot increase the risk of dryness more than that of OOD alone, and the difference was not statistically significant (RR = 14.18, 95% CI [0.10, 2051.73], *p* = 0.30, I^2^ = 88%) (Table 3).

**TABLE 3:** Subgroup analysis of adverse events in clinical studies.

References	Comparison	Experimental Group		Control Group		Risk Ratio, M-H, 95%CI	*p* value
		No. of Events	Total	No. of Events	Total		
**Diarrhea**							
Zhang, et al., 2018(a)	LD + OTT VS. OTT	2	30	5	30	0.36 [0.06, 2.01]	—
Zhang, et al., 2018(b)	LD + OTT VS. OTT	0	30	5	30	0.08 [0.00, 1.44]	—
Zhang 2020	LD VS. OOD	0	51	3	51	0.13 [0.01, 2.67]	—
Subtotal (95% CI) I^2^ = 0%		—	111	—	111	0.21 [0.06, 0.81]	*p* = 0.02
Total (95% CI) Random-effects I^2^ = 81%		—	201	—	201	0.98 [0.09, 11.10]	*p* = 0.98
**Dryness**							
Han 2006	LD + OOD VS. OOD	27	40	0	40	165.00 [9.41, 2892.45]	—
Luo, et al., 2018	LD + OOD VS. OOD	3	50	2	50	1.53 [0.24, 9.59]	—
Subtotal (95% CI) I^2^ = 88%		—	90	—	90	14.18 [0.10, 2051.73]	*p* = 0.30

#### 3.5.3 Skin Barrier Function

Two studies (Su, 2019; Gao et al., 2020) assessed skin barrier function from three perspectives: the water content of the stratum corneum, sebum content, and transepidermal water loss (TEWL). Compared with other drugs, CHM formulas with *Lithospermum erythrorhizon* as the sovereign herb significantly increased the content of water (MD = 5.55, 95% CI [4.14, 6.96], *p* < 0.00001, I^2^ = 0%) and sebum in the corneous layer (MD = 12.33, 95% CI [7.32, 17.33], *p* < 0.00001, I^2^ = 0%), although had no effect on TEWL (*p* = 0.32) (Table 4).

**TABLE 4:** Skin barrier function of included clinical studies.

References	Comparison	Change from baseline (Mean ± SD)		Mean Difference [95%CI]	*p* value
		Experiment	Control		
**The Water Content of the Stratum Corneum**					
Gao et al., 2020	LD + OTT VS. OTT	35.23 ± 4.08	29.45 ± 5.37	5.78 [3.81, 7.75]	—
Su et al., 2019	LT + OTT VS. OTT	54.34 ± 5.04	49.03 ± 5.26	5.31 [3.29, 7.33]	—
Total [95%CI] Random-effects I^2^ = 0%				5.55 [4.14, 6.96]	*p* < 0.00001
**the Sebum content**					
Gao et al., 2020	LD + OTT VS. OTT	146.78 ± 57.29	133.05 ± 41.84	13.73 [-7.00, 34.46]	—
Su et al., 2019	LT + OTT VS. OTT	143.03 ± 11.60	130.79 ± 14.54	12.24 [7.08, 17.40]	—
Total [95%CI] Random-effects I^2^ = 0%				12.33 [7.32, 17.33]	*p* < 0.00001
**TEWL(transepidermal water loss)**					
Gao et al., 2020	LD + OTT VS. OTT	0.12 ± 0.03	0.18 ± 0.04	−0.06 [−0.07, −0.05]	—
Su et al., 2019	LT + OTT VS. OTT	15.87 ± 4.22	19.87 ± 3.06	−4.00 [−5.44, −2.56]	—
Total [95% CI] Random-effects I^2^ = 96%				−1.96 [−5.82, 1.90]	*p* = 0.32

### 3.6 Primary Outcomes of Preclinical Studies

#### 3.6.1 PASI

A meta-analysis involving six studies (Wang et al., 2015; Zhao, 2016a; Wang et al., 2016; Yu et al., 2019; Zhang et al., 2019; Lan et al., 2020) assessed the efficacy of SHI or DMA in the treatment of psoriatic dermatitis in mice. Whether SHI or DMA, the meta-analyses results showed that they could significantly reduce the ear thickness (SHI group: MD = −1.74, 95% CI: 2.09 to −1.39, *p* < 0.00001; DMA group: MD = −0.63, 95% CI: 0.96 to −0.31, *p* = 0.0001) and PASI score (SHI group: MD = −4.57, 95%CI: 5.65 to −3.49, *p* < 0.00001; DMA group: MD = −1.71, 95% CI: 2.22 to −1.19, *p* < 0.00001) on day 7 of IMQ-induced psoriasis-like inflammation in mice. The specific terms of PASI score including erythema (SHI group: MD = −1.36, 95% CI: 1.92 to −0.79, *p* < 0.00001; DMA group: MD = −2.91, 95% CI: 4.98 to −0.85, *p* = 0.006) and scaling (SHI group: MD = −1.51, 95% CI: 1.99 to −1.04, *p* = 0.18; DMA group: MD = −0.42, 95% CI: 0.72 to −0.13, *p* = 0.005) were also down-regulated after treatment with SHI or DMA. They also had lower PASI scores (MD = −3.14, 95%CI: 4.92 − −1.35, *p* = 0.0006) on the 10th day. In summary, the results showed that SHI and DMA were more effective than conventional drugs in improving psoriatic dermatitis in mice (Table 5).

**TABLE 5:** Subgroup analysis of PASI scores in preclinical tstudies *in vivo*.

References	Change from Baseline (Mean ± SD)		Mean Difference [95%CI]	*p* value
	Experiment	Control		
**PASI(day 7)**				
**2.1.1 SHI**				
Lan et al.,2020	5.36 ± 1.32	11.40 ± 1.60	−6.04 [−7.86, −4.22]	—
Zhang et al., 2019-1	5.71 ± 0.73	9.09 ± 0.52	−3.38 [−4.17, −2.59]	
Zhang et al., 2019-2	5.01 ± 0.78	9.09 ± 0.52	−4.08 [−4.90, −3.26]	
Zhang et al., 2019-3	3.82 ± 0.26	9.09 ± 0.52	−5.27 [−5.78, −4.76]	
Subtotal (95% CI) I^2^ = 85%			−4.57 [−5.65, −3.49]	*p* < 0.00001
**2.1.2 DMA**				
Wang *et al*, 2015-1	3.13 ± 0.54	5.16 ± 1.02	−2.03 [−2.95, −1.11]	—
Wang *et al*, 2015-2	3.30 ± 0.32	5.16 ± 1.02	−1.86 [−2.72, −1.00]	
Wang *et al*, 2015-3	3.94 ± 0.51	5.16 ± 1.02	−1.22 [−2.13, −0.31]	
Subtotal (95% CI) I^2^ = 0%			−1.71 [−2.22, −1.19]	*p* < 0.00001
Total 95%CI Random-effects I^2^ = 94%			−3.36 [−4.67, −2.05]	*p* < 0.00001
**Erythema(day 7)**				
**2.2.1 SHI**				
Lan et al.,2020	1.84 ± 0.41	4.00 ± 0.23	−2.16 [−2.57, −1.75]	—
Zhang et al., 2019-1	2.04 ± 0.55	2.76 ± 0.28	−0.72 [−1.26, −0.18]	
Zhang et al., 2019-2	1.71 ± 0.25	2.76 ± 0.28	−1.05 [−1.38, −0.72]	
Zhang et al., 2019-3	1.31 ± 0.31	2.76 ± 0.28	−1.45 [−1.82, −1.08]	
Subtotal (95% CI) I^2^ = 87%			−1.36 [−1.92, −0.79]	*p* < 0.00001
**2.2.2 DMA**				—
Wang *et al*, 2015-1	1.09 ± 0.23	1.89 ± 1.08	−0.80 [−1.68, 0.08]	—
Wang *et al*, 2015-2	1.14 ± 0.33	5.16 ± 1.02	−4.02 [−4.88, −3.16]	—
Wang *et al*, 2015-3	1.24 ± 0.41	5.16 ± 1.02	−3.92 [−4.80, −3.04]	—
Subtotal (95% CI) I^2^ = 94%			−2.91 [−4.98, −0.85]	*p* = 0.006
Total 95%CI Random-effects I^2^ = 93%			−1.97 [−2.72, −1.22]	*p* < 0.00001
**Scaling(day 7)**				
**2.3.1 SHI**				
Lan et al.,2020	1.46 ± 0.57	3.78 ± 0.40	−2.32 [−2.93, −1.71]	—
Zhang et al., 2019-1	1.78 ± 0.37	2.85 ± 0.31	−1.07 [−1.49, −0.65]	
Zhang et al., 2019-2	1.68 ± 0.21	2.85 ± 0.31	−1.17 [−1.50, −0.84]	
Zhang et al., 2019-3	1.18 ± 0.38	2.85 ± 0.31	−1.67 [−2.10, −1.24]	
Subtotal (95% CI) I^2^ = 79%			−1.51 [−1.99, −1.04]	*p* < 0.00001
**2.3.2 DMA**				
Wang *et al*, 2015-1	0.91 ± 0.42	1.37 ± 0.34	−0.46 [−0.89, −0.03]	—
Wang *et al*, 2015-2	0.79 ± 0.23	1.37 ± 0.34	−0.58 [−0.91, −0.25]	
Wang *et al*, 2015-3	1.36 ± 0.64	1.37 ± 0.34	−0.01 [−0.59, 0.57]	
Subtotal (95% CI) I^2^ = 29%			−0.42 [−0.72, −0.13]	*p* = 0.005
Total 95%CI Random-effects I^2^ = 88%			−1.03 [−1.50, −0.56]	*p* < 0.0001
**Thickness(day 7)**				
**2.4.1 SHI**				
Lan et al.,2020	1.88 ± 0.80	3.47 ± 0.56	−1.59 [−2.45, −0.73]	—
Zhang et al., 2019-1	1.78 ± 0.39	3.27 ± 0.46	−1.49 [−2.02, −0.96]	
Zhang et al., 2019-2	1.70 ± 0.22	3.27 ± 0.46	−1.57 [−2.02, −1.12]	
Zhang et al., 2019-3	1.10 ± 0.19	3.27 ± 0.46	−2.17 [−2.61, −1.73]	
Subtotal (95% CI) I^2^ = 43%			−1.74 [−2.09, −1.39]	*p* < 0.00001
**2.4.2 DMA**				
Wang *et al*, 2015-1	1.09 ± 0.38	1.86 ± 0.58	−0.77 [−1.32, −0.22]	—
Wang *et al*, 2015-2	1.28 ± 0.45	1.86 ± 0.58	−0.58 [−1.17, 0.01]	
Wang *et al*, 2015-3	1.31 ± 0.37	1.86 ± 0.58	−0.55 [−1.10, 0.00]	
Subtotal (95% CI) I^2^ = 0%			−0.63 [−0.96, −0.31]	*p* = 0.0001
Total 95%CI Random-effects I^2^ = 82%			−1.25 [−1.74, −0.76]	*p* < 0.00001
**PASI(day 10)**				
Zhang et al., 2019-1	3.41 ± 1.18	4.02 ± 1.02	−0.61 [−1.63, 0.41]	—
Zhang et al., 2019-2	2.29 ± 0.98	4.02 ± 1.02	−1.73 [−2.65, −0.81]	—
Zhang et al., 2019-3	3.66 ± 0.81	6.66 ± 0.62	−3.00 [−3.89, −2.11]	—
Zhao 2016-1	2.16 ± 0.44	6.66 ± 0.62	−4.50 [−5.17, −3.83]	—
Zhao 2016-2	0.95 ± 0.37	6.66 ± 0.62	−5.71 [−6.34, −5.08]	—
Total 95%CI Random-effects I^2^ = 96%			−3.14 [−4.92, −1.35]	*p* = 0.0006

### 3.7 Secondary Outcomes of Clinical Studies

#### 3.7.1 Epidermal Thickness

Compared with the control group, three studies (Wang et al., 2015; Zhao, 2016a; Yu et al., 2019) showed that SHI and DMA were effective in reducing the epidermal thickness of psoriatic lesions in mice (MD = −34.42, 95% CI: 41.25 to −27.59, *p* < 0.00001). We then performed subgroup analysis based on the two components of *Lithospermum erythrorhizon*, the results showed that both SHI and DMA reduced the epidermal thickness of mice and had a certain therapeutic effect on psoriasis (SHI group: MD = −33.91, 95% CI: 43.39 to −24.43, *p* < 0.00001; DMA group: MD = −35.04, 95% CI: 47.66, −22.42, *p* < 0.00001) (Table 6).

**TABLE 6:** Subgroup analysis of epidermal thickness in preclinical studies *in vivo*.

References	Change from Baseline (Mean ± SD)		Mean Difference [95%CI]	*p* value
	Experiment	Control		
**Epidermal thickness**				
**3.2.1 SHI**				
Yu *et al*, 2019-1	47.76 ± 5.53	70.34 ± 7.06	−22.58 [−29.76, −15.40]	—
Yu *et al*, 2019-2	32.18 ± 2.76	70.34 ± 7.06	−38.16 [−44.23, −32.09]	
Zhao 2016-1	42.55 ± 2.35	72.16 ± 4.85	−29.61 [−33.13, −26.09]	
Zhao 2016-2	27.79 ± 1.16	72.16 ± 4.85	−44.37 [−47.63, −41.11]	
Subtotal (95% CI)			−33.91 [−43.39, −24.43]	*p* < 0.00001
**3.2.2 DMA**				
Wang *et al*, 2015-1	31.30 ± 1.43	78.26 ± 5.64	−46.96 [−51.62, −42.30]	—
Wang *et al*, 2015-2	45.46 ± 2.60	78.26 ± 5.64	−32.80 [−37.77, −27.83]	
Wang *et al*, 2015-3	53.10 ± 4.01	78.26 ± 5.64	−25.16 [−30.70, −19.62]	
Subtotal (95% CI)			−35.04 [−47.66, −22.42]	*p* < 0.00001
Total 95%CI Random-effects I^2^ = 93%			−34.42 [−41.25, −27.59]	*p* < 0.00001

#### 3.7.2 IL-17A

Two studies (Wang et al., 2015; Zhao, 2016a) have reported the expression levels of IL-17A. Meta-analysis showed that SHI reduced IL-17A levels in psoriatic lesions of mice (MD = −2.71, 95% CI: 3.81 to −1.60, *p* < 0.00001) (Table 7).

**TABLE 7:** IL-17A of included preclinical studies *in vivo*.

References	Change from Baseline (Mean ± SD)		Mean Difference [95%CI]	*p* value
	Experiment	Control		
Zhang, 2019-1	0.86 ± 0.20	2.64 ± 0.45	−1.78 [−2.21, −1.35]	—
Zhang, 2019-2	0.77 ± 0.10	2.64 ± 0.45	−1.87 [−2.27, −1.47]	
Zhang, 2019-3	0.58 ± 0.11	2.64 ± 0.45	−2.06 [−2.47, −1.65]	
Zhao, 2016-1	3.45 ± 0.39	6.83 ± 0.36	−3.38 [−3.73, −3.03]	
Zhao, 2016-2	2.41 ± 0.25	6.83 ± 0.36	−4.42 [−4.71, −4.13]	
Total 95%CI Random-effects I^2^ = 98%			−2.71 [−3.81, −1.60]	*p* < 0.00001

## Discussion

### Summary of Evidence

This is the first systematic review of the clinical efficacy, safety, and potential mechanism of action of *Lithospermum erythrorhizon* and its active components in the treatment of psoriasis. Evidence from 12 clinical trials involving 1,024 participants showed that CHM formulas with *Lithospermum erythrorhizon* as the sovereign herb were more effective in the treatment of psoriasis. Specifically, LD was better than OOD in improving the effectiveness in patients with psoriasis. The combined application of LD and OOD was better than that of OOD alone, and the combined use of LT and OTT improved the efficacy. In preclinical studies, six animal experiments involving 185 mice and 17 cell experiments involving 5 cell types demonstrated that SHI and DMA have therapeutic effects in animal models of psoriasis, possibly exerting anti-inflammatory effects by promoting keratinocyte apoptosis, inhibiting keratinocyte proliferation and angiogenesis, and blocking the cell cycle.

### Limitations

CHM formulations used in clinical research lack valid assessments, toxicological studies, pharmacologic studies, quality control of each component, product information of the formulas, safety assessments, and authentication methods. Because the composition of CHM formulations are very complex, it is difficult to conduct efficacy and toxicological studies. Moreover, the sample size was small, and the conclusions drawn may not be sufficiently reliable. The quality of the trials included in this meta-analysis was not very high. Although all these studies introduced random methods, none were designed to be blinded by researchers, participants, and statisticians. Finally, this study included unpublished master’s and doctoral dissertations.

### Implications

This systematic review included 34 studies, including 11 clinical studies and 23 preclinical studies, which evaluated the efficacy and safety of *Lithospermum erythrorhizon* and its active components in the treatment of psoriasis and explained the mechanism of SHI and DMA in the treatment of psoriasis through *in vivo* and *in vitro* experiments. The results showed that CHM formulas with *Lithospermum erythrorhizon* as the sovereign herb could significantly improve the PASI score. Although *Lithospermum erythrorhizon* is effective in treating psoriasis, its side effects cannot be ignored. TCM believes that psoriasis is mostly caused by blood, heat, and toxins (Guideline for the diagnosis and, 2019); therefore, the treatment often uses TCM that clears heat and cools blood, promotes blood circulation, and removes blood stasis (Sun et al., 2020). However, this type of Chinese medicine can easily affect the spleen and stomach, which may be the cause of diarrhea. Therefore, it is recommended that some drugs be added to protect the spleen and stomach when using Chinese medicine to treat psoriasis. Excessive proliferation of epidermal keratinocytes in psoriasis damages the skin barrier function to varying degrees and reduces the skin’s water-locking and anti-inflammatory functions (Montero-Vilchez, 2021). CHM formulas with *Lithospermum erythrorhizon* as a sovereign herb can effectively improve the clinical symptoms of patients with psoriasis vulgaris, increase skin keratin water content and sebum content, and reduce TEWL to improve skin barrier and immunity functions (Su, 2019; Gao et al., 2020).

TCM has achieved excellent results in the treatment of diseases in mainland China, Taiwan, and Japan. However, due to the lack of standards, systematic explanations, and explanations of modern sciences, such as physiology, chemistry, and pharmacology, it is difficult to promote it to the world. However, in recent years, with increasing attention on TCM, it has gradually begun to become standardized in modern research. As proof, Tu Youyou, winner of the 2015 Nobel Prize in Physiology or Medicine, isolated artemisinin from CHM and used it to treat malaria. This research achievement has saved millions of lives and halved the mortality rate of malaria (Tiwari and Chaudhary, 2020). Wang Chen et al. used standardized and strict modern evidence-based medical research methods to prove that the TCM decoction, Maxing Shigan decoction, and Yinqiaosan modified decoction are similar to or better than Tamiflu in the treatment of new type A H1N1 influenza. This research is internationally recognized (Wang, 2011). In future research, we need to pay more attention to the standardization of TCM, and strictly follow the list of CHM formulas.Preclinical research helps us better explore the pathogenic mechanism. Psoriasis is considered an autoimmune skin disease in which the keratinocytes (KC)/DC/T loop plays a major role (Liu et al., 2021). In psoriasis, DCs secrete cytokines such as IL-23 to activate downstream T cells, and activated T cells secrete pro-inflammatory factors such as IL-17 to act on KCs, leading to psoriatic inflammation (Sato et al., 2020). SHI and DMA affected the cascade reaction of the entire loop. First, it inhibits KC proliferation, promotes apoptosis, and inhibits proinflammatory factors and antimicrobial peptides secreted by KCs to reduce psoriasis. Furthermore, it can reduce DC activity and DC-related inflammatory factors to ameliorate psoriasis. Additionally, it can reduce the activation of T-cells and their secretion of pro-inflammatory factors, such as IL-17, to improve psoriasis. Skewness in Thl cell differentiation has been demonstrated in psoriasis (Wang, 2017). SHI and DMA can also affect Th1 cell activity and TH1 and TH2 cell balance to improve psoriasis (Zhang, 2011). In this study, we found that SHI exerts therapeutic effects on HaCaT cells, DCs, and T cells. SHI may inhibit HaCaT cell proliferation and promote apoptosis by blocking the G0/G1 phase and reducing the cell distribution in the S and G2/M phases (Yu et al., 2019; Wang et al., 2011) These effects may be achieved by inhibiting the JAK/STAT3, JNK2/MAPK, or ERK1/2/MAPK signaling pathways to affect CCAAT/enhancer binding protein delta, GRB2-associated binding protein 1 (Gab1) and GRB2-associated binding protein 2 (Gab2), and other downstream genes (Yu et al., 2019; Lan et al., 2020; Zhu, 2013; Zhao, 2016b). Moreover, the expression of inflammatory cytokines such as vascular endothelial growth factor (VEGF), IL-17, IL-6, and IL-23 in keratinocytes was inhibited (Xing, 2010; Xu et al., 2014), while the levels of chemokines, including CXCL1, CXCL2, and CCL20 (Xie, 2015) were reduced. Antimicrobial peptides secreted by keratin-forming cells, including S100A7 and S100A8, were also reduced by treatment with SHI (Zhu, 2013). Two studies focused on DCs. SHI and DMA inhibited the expression of molecules on the surface of DCs, such as CD86, CD83, and CD86 (Wang et al., 2016; Wang, 2014). IL-23 expression was also inhibited. Furthermore, DMA mainly acts by decreasing the expression of TLR7, MyD88, and IRAKM in DCs (Wang et al., 2016). The included articles suggested that Th1 and Th2 cells may be key targets for SHI and DMA treatment. For human PBMCs, Zhang et al. (Zhang, 2011) demonstrated that SHI may inhibit IFN-γ secretion by affecting T-cell activity, especially Th1 cell activation, and in turn, increase Th2 cytokine secretion. SHI can also inhibit IL-6 and IL-17 production by PBMCs induced by IL-23 in patients with psoriasis. Liu *et al.* (Liu, 2018) observed SHI-treated Jurkat E6-1 cells and demonstrated that SHI can inhibit the mRNA and protein expression of nuclear transcription factors such as NF-AT, AP-1, and NF-κB by downregulating the concentration of the second messenger (Ca2^+^) and the level of key node kinase PKC in the signal transduction pathway. Thus, the signal transduction pathway involved in T-lymphocyte activation can be regulated. Based on the above studies, we summarized the mechanisms of psoriasis treatment with SHI and DMA (Supplementary Table S3). B-lymphocytes are also crucial for the pathogenesis of psoriasis. Activated B lymphocytes are upregulated in the peripheral blood of patients with psoriasis compared to those in healthy donors (Niu et al., 2015). In IMQ-induced dermatitis, mice lacking B cells showed a stronger inflammatory response (Alrefai et al., 2016). In this study, we did not find studies on SHI and DMA treatment of psoriasis through B cells; hence, our research mainly focused on T cells. The underlying mechanisms of SHI and DMA in psoriasis treatment are summarized in Figure 1. Although the quality of the articles included in the study was not satisfactory, the safety and efficacy of *Lithospermum erythrorhizon* for psoriasis treatment have been proven. In addition, it clarified the potential mechanism of *the active components of Lithospermum erythrorhizon* in psoriasis treatment. To further study the efficacy of *Lithospermum erythrorhizon* in the treatment of psoriasis, clarify its mechanism, and confirm the results of this quantitative study, a large number of high-quality clinical and preclinical trials with low bias and sufficient sample sizes are required in the future.

## Conclusion

In conclusion, the TCM prescription that incorporates *Lithospermum erythrorhizon* as the sovereign herb has better efficacy in the treatment of psoriasis, and on this basis, LD has a better curative effect than OOD. The combination of LD and OOD and the combination of LT and OTT can significantly improve the curative effect. Moreover, the results of preclinical trials showed that the active components of *Lithospermum erythrorhizon*, SHI, and DMA have potential anti-inflammatory effects, promote keratinocyte apoptosis, inhibit keratinocyte proliferation and angiogenesis, and block the cell cycle. Furthermore, SHI and DMA can affect DC and cell activity to achieve therapeutic effects.

## Data Availability

The original contributions presented in the study are included in the article/Supplementary Material, further inquiries can be directed to the corresponding author.
