# Supplementary figures and images for: Evidence and Potential Mechanism of Action of Lithospermum erythrorhizon and Its Active Components for Psoriasis
Source: Front Pharmacol. 2022 May 5;13:781850. doi: 10.3389/fphar.2022.781850 (PMC9128614; doi:10.3389/fphar.2022.781850)

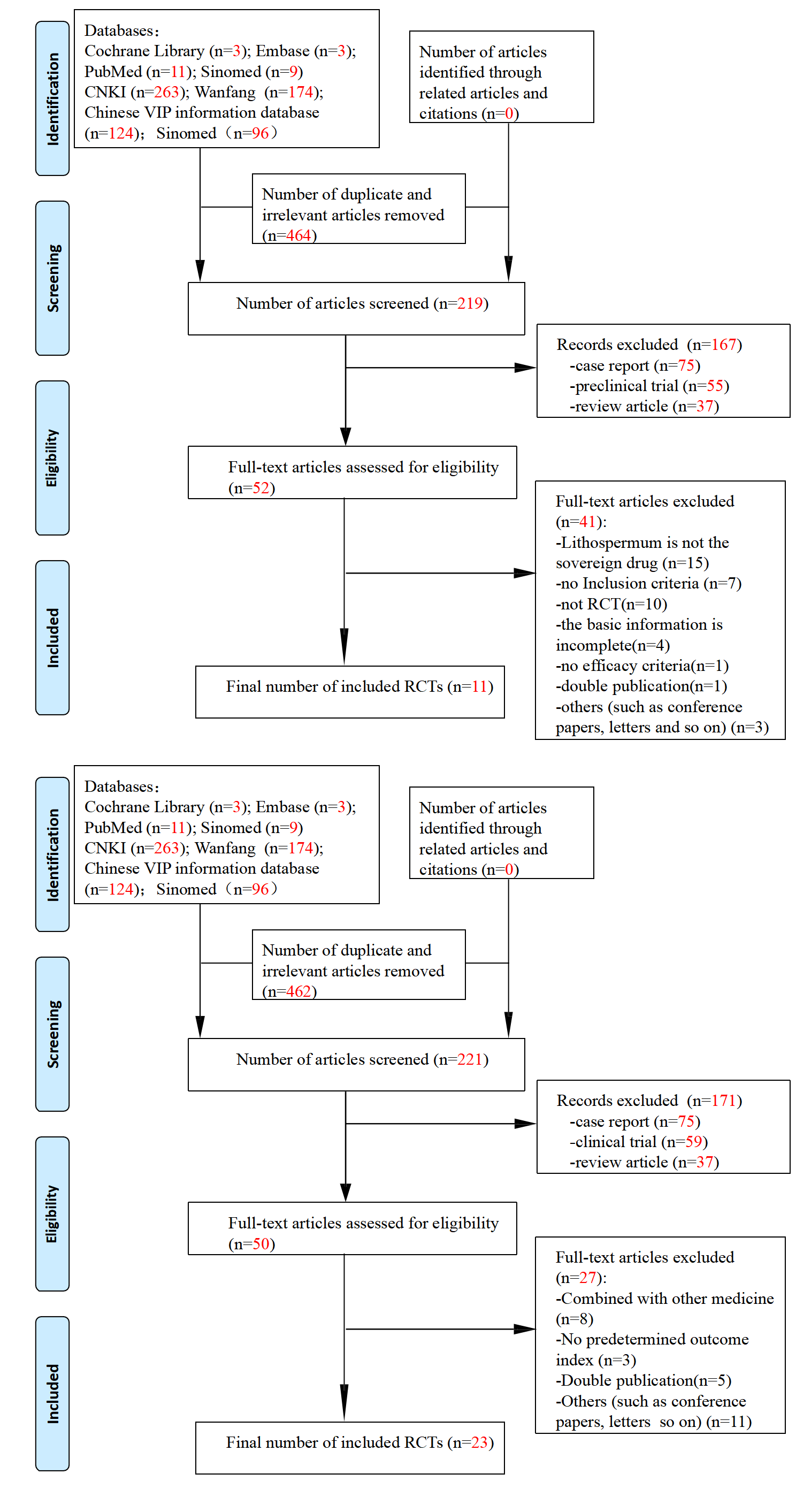

Supplement: Supplementary file 1 [file Image1.tiff]

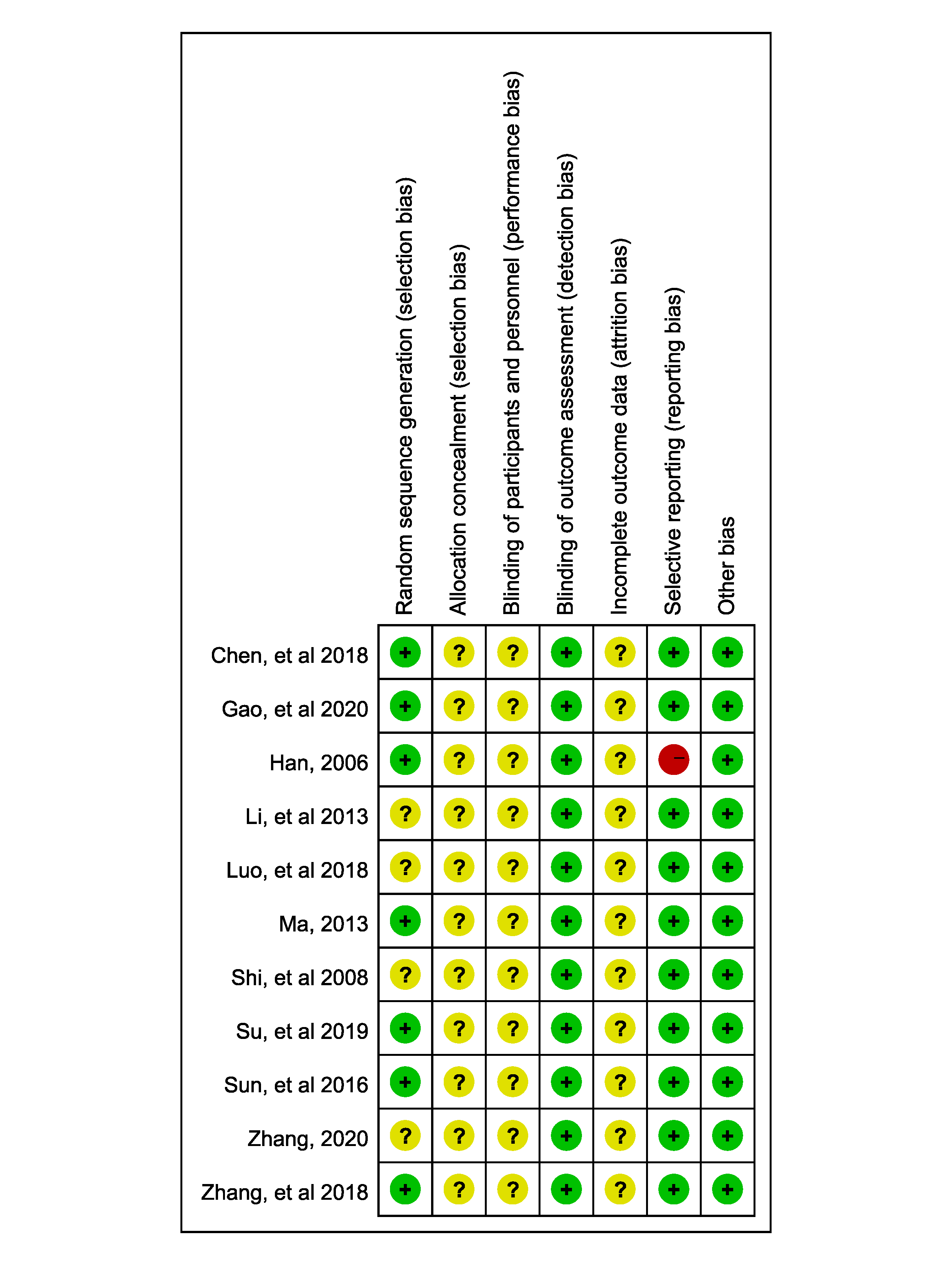

Supplement: Supplementary file 3 [file Image3.tif]

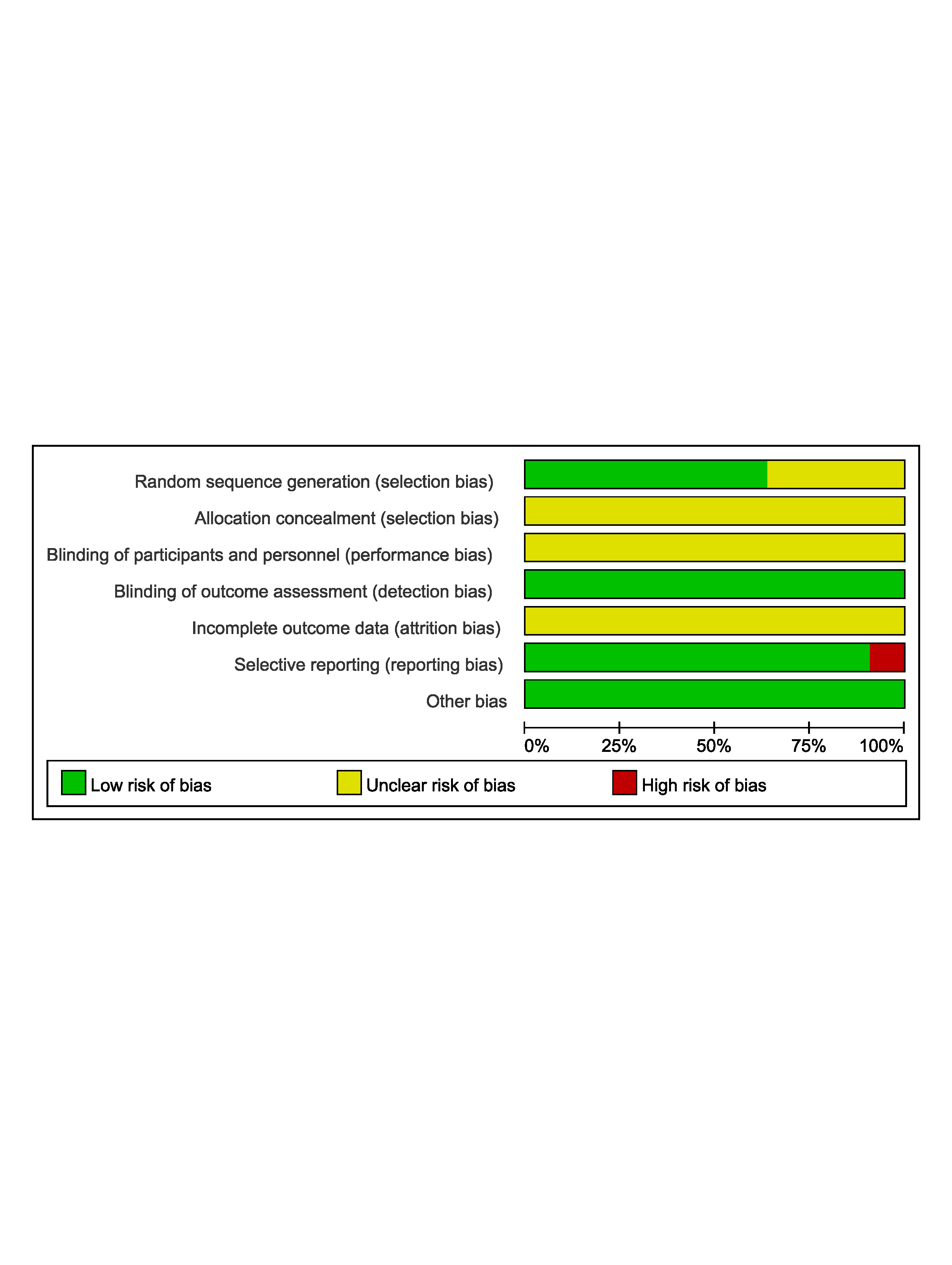

Supplement: Supplementary file 8 [file Image2.tiff]

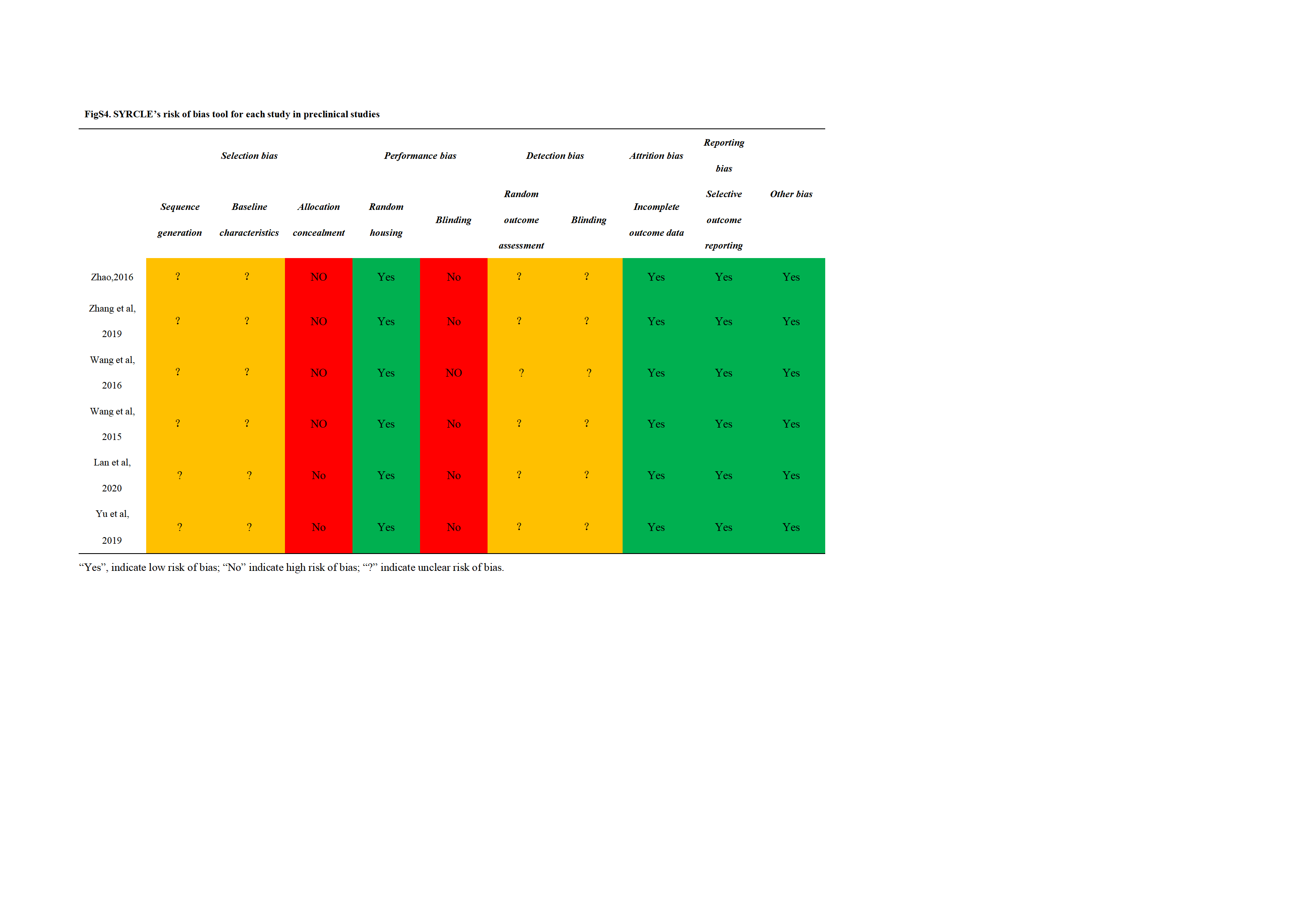

Supplement: Supplementary file 9 [file Image4.tiff]
