# Supplementary material for: Evidence and Potential Mechanism of Action of Lithospermum erythrorhizon and Its Active Components for Psoriasis
Source: Front Pharmacol. 2022 May 5;13:781850. doi: 10.3389/fphar.2022.781850 (PMC9128614; doi:10.3389/fphar.2022.781850)
Supplement: Supplementary file 2 [file Table1.docx]

| **Table S1. Checklist of Items for Reporting Trials of Chinese Herbal Medicine Formulas** | | | | | |  |  |  |  |  |  |  |  |  |  |
| --- | --- | --- | --- | --- | --- | --- | --- | --- | --- | --- | --- | --- | --- | --- | --- |
| **Category** | **Study** | **Chinese medicine compound** | **Composition and dosage of Chinese medicine prescriptions (Chinese Pinyin name and Latin name)** | **Authentication method** | **Principles, rationale, and interpretation of**  **forming the formula** | **Reference(s) as to the efficacy of the formula** | **Pharmacologic study results of the formula** | **Production method of the formula** | **Quality control of each ingredient and of the**  **product of the formula** | **Safety assessment of the formula** | **Dosage of the formula** | **Administration Route** | | | **Preparation method** |
| Clinical research | Han 2006 | Zilian Decoction | Zicao (*Lithospermum erythrorhizon*) 15 g  Lianqiao (*Forsythiae Fructus*) 9 g  Qinjiao (*Gentiana Macrophylla Pall*) 9 g  Chishao (*Radix Paeoniae Rubra*) 15 g  (*Carthami Flos*) 6 g  Wumei (*Mume Fructus*) 30 g  Ezhu (*Curcumae Rhizoma*) 9 g  Gancao (*licorice*) 12 g  Difuzi (*Kochiae Fructus*) 15 g  Shengmuli (*raw oyster*) 30 g | N/A | Zicao, Lianqiao, Qinjiao, Chishao, Honghua,Wumei, Ezhu, Difuzi: inhibit the rapid growth of epidermal cells, anti-inflammatory.  Shengmuli: improve immunity.  Gancao: antibacterial, anti-inflammatory, hypoglycemic, lipid-lowering, antioxidant, anti-allergic. | [19] | N/A | N/A | N/A | N/A | bid, 12 w | Oral | | | Decoction |
|  | Shi, et al. 2008 | Liangxue Jiedu Decoction | Zicao (*Lithospermum erythrorhizon*) 10 g  Mudanpi (*Cortex Moutan*) 10 g  Chishao (*Radix Paeoniae Rubra*) 10 g  Danshen (*Radix Salviae*) 10 g  Jixueteng (*Spatholobus Suberectus Dunn*) 10 g  Dihuang (*Radix Rehmanniae*) 30 g  Baimaogen (*Imperatae Rhizoma*) 30 g  Tufulin (*Smilacis Glabrae Rhixoma*) 30 g | N/A | Zicao, Chishao, Baimaogen: anti-inflammatory.  Mudanpi: anti-inflammatory, anti-allergic and immunomodulatory effects.  Danshen: improve blood rheology, improve microcirculation, improve hemodynamics, promote tissue repair and regeneration.  Dihuang: antibacterial, anti-inflammatory, enhances immune function, lowers blood sugar.  Tufulin: antibacterial and anti-inflammatory effects. | [20] | N/A | N/A | N/A | N/A | bid, 8 w | Oral | | | Decoction |
|  | Li, et al 2013 | Mahuang Zimei Decoction | Mahuang (*Ephedra Herba*) 10 g  Guizhi (*Cinnamomi Ramulus*) 12 g  Shengdihuang (*Radix Rehmanniae*) 20 g  Chishao (*Radix Paeoniae Rubra*) 12 g  Shashen (*Adenophprae Ae Radix*) 10 g  Zicao (*Lithospermum erythrorhizon*) 15 g  Qiancao (*Rubia Cordifolia*) 15 g  Baihuasheshecao (*Hedyotis Diffusae Herba*) 12 g  Wumei (*Mume Fructus*) 30 g  Tufulin (*Smilacis Glabrae Rhixoma*) 30 g  Baqia (*Smilax China*) 30g | N/A | Mahuang, Guizh, Chishao, Zicao, Qiancao, Wumei: anti-inflammatory.  Shengdihuang: antibacterial, anti-inflammatory, enhances immune function, lowers blood sugar.  Shashen: immune regulation.  Baihuasheshecao, Tufulin, Baqia: antibacterial and anti-inflammatory effects. | [21] | N/A | N/A | N/A | N/A | bid, 300 ml, 4 w | Oral | | | Decoction |
|  | Luo, et al 2018 | Zicao Huoxue Decoction | Zicao (*Lithospermum erythrorhizon*) 15 g  Banxia (*Arum Ternatum Thunb*) 10 g  Huanglian (*Coptidis Rhizoma*) 10 g  Tubiechong (*Eupolyphaga*) 10 g  Chantui (*Cicadae Periostracum*) 10 g  Mudanpi (*Cortex Moutan*) 10 g  Qindai (*Indigo Naturalis*) 10 g  Chenpi (*Citrus Reticulata*) 10 g  Houpo (*Magnolia Officinalis Rehd Et Wils.*) 10g, Difuzi (*Kochiae Fructus*) 10 g  Baixianpi (*Dictamni Cortex*) 10 g  Danggui (*Angelicae Sinensis Radix*) 10 g  Dilong (*Pheretima*) 15 g | N/A | Zicao, Banxia, Qindai: anti-inflammatory.  Huanglian, Tubiechong, Chantui, Dilong: anti-inflammatory, lipid-lowering, and hypoglycemic effects.  Mudanpi: anti-inflammatory, anti-allergic, and immunomodulatory effects.  Chenpi: antioxidant, lipid lowering, anti-inflammatory  Houpo, Baixianpi: anti-inflammatory, analgesic, antibacterial, antioxidant.  Danggui: anti-inflammatory, immune regulation | [24] | N/A | N/A | N/A | N/A | bid, 200 ml, 8 w | Oral | | | Add 2,000 mL of water to decoct the traditional Chinese medicine compound 1.5 hours to 400 mL of the remaining decoction. |
|  | Zhang, et al 2018 | Zicao Decoction | Zicao (*Lithospermum erythrorhizon*) 30 g  Dihaung (*Rehmanniae Radix Praeparata*) 30 g  Gancao (*licorice*) 12 g | N/A | Zicao: anti-inflammatory.  Dihaung: antibacterial, anti-inflammatory, enhances immune function, lowers blood sugar.  Gancao: antibacterial, anti-inflammatory, hypoglycemic, lipid-lowering, antioxidant, anti-allergic. | [26] | N/A | N/A | N/A | N/A | bid, 8 w | Oral | | | Decoction |
|  | Gao, et al 2020 | Zicao Biejia Siwu Decoction | Zicao (*Lithospermum erythrorhizon*) 20 g  Biejia (*Carapax Trionycis*) 15 g  Dihuang (*Radix Rehmanniae*) 15 g  Danggui (*Angelicae Sinensis Radix*) 10 g  Baishao (*Paeoniae Radix Alba*) 10 g  Chuanxiong (*Chuanxiong Rhizoma*) 10 g | N/A | Zicao, Baishao: anti-inflammatory.  Biejia, Chuanxiong: immune regulation.  Dihaung: antibacterial, anti-inflammatory, enhances immune function, lowers blood sugar.  Danggui: anti-inflammatory, immune regulation. | [28] | N/A | N/A | N/A | N/A | bid, 250 ml, 8 w | Oral | | | Soak the traditional Chinese medicine in 500ml of water for 4 hours, boil over high heat until it boils, and then boil over low heat for 30 minutes. |
|  | Zhang 2020 | Zicao Decoction | Dihaung (*Rehmanniae Radix Praeparata*) 30 g  Baimaogen (*Imperatae Rhizoma*) 30 g  Zicao (*Lithospermum erythrorhizon*) 30 g  Shuiniujiao (*Buffalo Horn*) 15 g  Banlangen (*Isatidis Radix*) 15 g  Jinyinhua (*Lonicerae Japonicae Flos*) 15 g  Daqingye (*Isatidis Folium*) 15 g  Chishao (*Radix Paeoniae Rubra*) 10 g  Mudanpi (*Cortex Moutan*) 10 g  Huaihua (*Sophora Japonica L.*) 10 g  Zhizi (*Gardeniae Fructus*) 10 g  Huangqin (*Scutellariae Radix*) 10 g | N/A | Dihaung: antibacterial, anti-inflammatory, enhances immune function, lowers blood sugar.  Zicao, Baimaogen, Zhizi, Huangqin: anti-inflammatory.  Shuiniujiao: sedative effect.  Banlangen: immune regulation.  Jinyinhua, Daqingye: anti-inflammatory, boosts immunity.  Mudanpi: anti-inflammatory, anti-allergic, and immunomodulatory effects.  Huaihua: Lowers blood pressure, dilates coronary arteries. | [29] | N/A | N/A | N/A | N/A | bid, 4 w | Oral | | | Decoction |
|  | Sun, et al 2016 | Zicao Oil | Zicao (*Lithospermum erythrorhizon*)  Mayou (*Sesame oil*) | N/A | Zicao: anti-inflammatory. | [23] | N/A | N/A | N/A | N/A | Moderate, 4 w | external use | | | Soak the zicao in sesame oil for 1 week (the amount of sesame oil is just less than the comfrey), then deep fry the comfrey until the comfrey is scorched, turn off the fire and filter out the drug residues. |
|  | Su, et al 2019 | Zicao Oil | Zicao (*Lithospermum erythrorhizon*)  Danggui: *Angelicae Sinensis Radix*  Baishao: *Paeoniae Radix Alba*  Baizhi*: [A. Dahurica (Fisch.) Benth. Et Hook](https://old.tcmsp-e.com/tcmspsearch.php?qr=A.%20Dahurica%20(Fisch.)%20Benth.%20Et%20Hook&qsr=herb_en_name&token=4a4ab5a4bdfa219c31e4fe9801a0a2f0)*  Honghua: *[Carthami Flos](https://old.tcmsp-e.com/tcmspsearch.php?qr=Carthami%20Flos&qsr=herb_en_name&token=4a4ab5a4bdfa219c31e4fe9801a0a2f0)*  Mayou (*Sesame oil*) | N/A | Zicao, Baishao, Baizhi: anti-inflammatory.  Dihaung: antibacterial, anti-inflammatory, enhances immune function, lowers blood sugar.  Honghua: inhibits the rapid growth of epidermal cells, anti-inflammatory. | [27] | N/A | N/A | N/A | N/A | moderate, 4 w | external use | | | Combine Zicao, Danggui, Baizhi, and Honghua a 2:1:1:1 ratio, fry in edible sesame oil until browned, remove residue and melt with beeswax. |
|  | Ma 2013 | Zicao Ointment | Zicao (*Lithospermum erythrorhizon*)  Danggui: *Angelicae Sinensis Radix*  Baishao: *Paeoniae Radix Alba*  Baizhi*: [A. Dahurica (Fisch.) Benth. Et Hook](https://old.tcmsp-e.com/tcmspsearch.php?qr=A.%20Dahurica%20(Fisch.)%20Benth.%20Et%20Hook&qsr=herb_en_name&token=4a4ab5a4bdfa219c31e4fe9801a0a2f0)*  Honghua: *[Carthami Flos](https://old.tcmsp-e.com/tcmspsearch.php?qr=Carthami%20Flos&qsr=herb_en_name&token=4a4ab5a4bdfa219c31e4fe9801a0a2f0)*  Mayou (*Sesame oil*) |  | Zicao, Baishao, Baizhi: anti-inflammatory.  Dihaung: antibacterial, anti-inflammatory, enhances immune function, lowers blood sugar.  Honghua: inhibits the rapid growth of epidermal cells, anti-inflammatory. | [22] | N/A | N/A | N/A | N/A | moderate, 4 w | external use | | | Combine Zicao, Danggui, Baizhi, and Honghua a 2:2:1:1 ratio, fry in edible sesame oil until browned, remove residue and melt with beeswax. |
|  | Chen, et al 2018 | Zicao Quyin Lotion | Zicao (*Lithospermum erythrorhizon*) 30 g  Shuiniujiao (*Buffalo Horn*) 20 g  Difuzi (*Kochiae Fructus*) 20 g  Dihuang (*Rehmanniae Radix Praeparata*) 20 g  Tufulin (*Smilacis Glabrae Rhixoma*) 20 g  Baixianpi (*Dictamni Cortex*) 20 g  Jinyinhua (*Lonicerae Japonicae Flos*) 15 g  Pugongying (*Taraxacum erpyhropodium kitag*) 15 g | N/A | Zicao: anti-inflammatory.  Shuiniujiao: sedative effect.  Dihaung: antibacterial, anti-inflammatory, enhances immune function, lowers blood sugar.  Dihuang: antibacterial, anti-inflammatory, enhances immune function, lowers blood sugar.  Tufulin: antibacterial and anti-inflammatory effects.  Jinyinhua, Daqingye: anti-inflammatory, boosts immunity.  Baixianpi: anti-inflammatory, analgesic, antibacterial, and antioxidant effects. | [25] | N/A | N/A | N/A | N/A | qd, 500 ml, 4 w | external use | | | Decoction in the hospital, each pack is 500 ml. |

**Abbreviations:** TCM, traditional Chinese medicine; N/A, not applicable; w, weeks; qd, once daily; bid, twice daily.
