# Supplementary material for: Evidence and Potential Mechanism of Action of Lithospermum erythrorhizon and Its Active Components for Psoriasis
Source: Front Pharmacol. 2022 May 5;13:781850. doi: 10.3389/fphar.2022.781850 (PMC9128614; doi:10.3389/fphar.2022.781850)
Supplement: Supplementary file 4 [file Table2.docx]

| **Table S2.the characteristics of included clinical studies** | | | | | | | | | | | | | | |
| --- | --- | --- | --- | --- | --- | --- | --- | --- | --- | --- | --- | --- | --- | --- |
| **Author Year** | **Sample Size** | | **Average Age (years) (Mean±SD)** | | **Gender(M/F)** | | **Duration of Psoriasis (year) (Mean±SD)** | | **Intervention** | | **Course of  treatment（week）** | **0utcome** | **Adverse  events** | |
|  | **E** | **C** | **E** | **C** | **E** | **C** | **E** | **C** | **E** | **C** |  |  | **E** | **C** |
| Han 2006 | 40 | 40 | 35 | 38 | 21/19 | 23/17 | 10.50 | 8.60 | LD + OOD | OOD | 12 | Efficiency, AEs | 29 | 0 |
| Shi, *et al* 2008 | 105 | 36 | 38.80 | 37.20 | 65/40 | 22/14 | 5.40 | 4.80 | LD + OTT | OOD + OTT | 8 | PASI, Efficiency | N/A | N/A |
| Li, *et al* 2013 | 60 | 60 | 35.24±10.28 | 33.48±10.02 | 34/26 | 32/28 | 6.50±4.10 | 5.40±3.70 | LD + OTT | OOD + OTT | 12 | PASI, Efficiency | N/A | N/A |
| Ma 2013 | 44 | 39 | 38.50 | 35 | 24/20 | 18/21 | 4 | 2 | LT + OOD | OOD | 4 | Efficiency, AEs | 3 | 1 |
| Sun, *et al* 2016 | 30 | 28 | 54.70±1.50 | 48.60±2.30 | 7/23 | 10/18 | 9.80±1.80 | 11.00±2.30 | LT + OOD | OOD | 4 | PASI, Efficiency | N/A | N/A |
| Luo, *et al* 2018 | 50 | 50 | 32.46±10.24 | 33.57±10.82 | 31/19 | 29/21 | 2.46±1.24 | 2.52±1.28 | LD + OOD | OOD | 8 | Efficiency, AEs | 10 | 8 |
| Chen, *et al* 2018 | 30 | 30 | 36.70±10.80 | 38.90±12.10 | 16/14 | 17/13 | 4.12±1.75 | 3.97±1.32 | LT | OTT | 4 | PASI, Efficiency, AEs | 1 | 4 |
| Zhang, *et al* 2018 | E1:30 | 30 | N/A | N/A | N/A | N/A | N/A | N/A | E1:LD (high)  + OTT | OTT | 8 | PASI, Efficiency, AEs | 2 | 5 |
|  | E2:30 |  | N/A |  | N/A |  | N/A |  | E2:LD (low)  + OTT |  |  |  | 0 |  |
| Su, *et al* 2019 | 50 | 50 | 51.50±4.90 | 51.80±4.40 | 36/14 | 34/16 | 2.20±0.90 | 1.90±0.60 | LT + OTT | OTT | 4 | PASI, Efficiency, Indicators of skin barrier function(the sebum content、 TEWL、the water content of the stratum corneum) | N/A | N/A |
| Gao, *et al* 2020 | 45 | 45 | 30.67± 5.65 | 30.61±5.74 | 24/21 | 23/22 | 3.33±1.27 | 3.38±1.21 | LD + OTT | OTT | 8 | Efficiency, Indicators of skin barrier function(the sebum content、 TEWL、the water content of the stratum corneum) | N/A | N/A |
| Zhang 2020 | 51 | 51 | 44.17±5.62 | 42.73±5.81 | 15/26 | 24/27 | N/A | N/A | LD | OOD | 4 | Efficiency, AEs | 2 | 12 |

**Abbreviations：**AE, Adverse Events; VAS, Visual Analogue Scales; E, Experiment; C, Control; TCM, Traditional Chinese Medicine; CHM, Chinese herbal medicine; NB-UVB, narrow band ultraviolet; PASI, psoriasis area and severity index; TEWL, transepidermal water loss; N/A, Not applicable; BP, blank group; M, Male; F, Female; w, weeks; m, months; y, years; LD (Decoction containing *Lithospermum erythrorhizon*: CHM Formulas with *Lithospermum erythrorhizon* as the sovereign herb include Zilian Decoction, Liangxue Jiedu Decoction, Mahuang Zimei Decoction, Zicao Huoxue Decoction, Zicao Decoction, Zicao Biejia Siwu Decoction); LT (topical CHM formulas containing Lithospermum erythrorhizon: Ointment, oil or lotion with Lithospermum erythrorhizon as the main herb, include Zicao ointment, Zicao oil, Zicao Quyin Lotion); OOD, other oral drugs(include Diyin tablets, Compound aminopeptin tablets, Liangxue Huoxue Decotion, TCM, Acitretin, Tripterygium wilfordii tablets); OTT, other topical therapies (include Vaseline, Pulian ointment, Capitriol ointment, TCM steam therapy, NB-UVB, Tacrolimus ointment).
