# Supplementary material for: Evidence and Potential Mechanism of Action of Lithospermum erythrorhizon and Its Active Components for Psoriasis
Source: Front Pharmacol. 2022 May 5;13:781850. doi: 10.3389/fphar.2022.781850 (PMC9128614; doi:10.3389/fphar.2022.781850)
Supplement: Supplementary file 5 [file Table5.docx]

| **Table S5. Checklist of Lithospermum erythrorhizon and its main components** | | | | | | |  |  |
| --- | --- | --- | --- | --- | --- | --- | --- | --- |
| **Category** | | **Study** | **Researched Medicine** | **Access** | **Route of administration** | **Experimental concentration** | **Dose** | **Preparation method** |
| Preclinical research | *in vivo* | Wang et al, 2015 | DMA | National Institute for the Control of Pharmaceutical and Biological Products | i.g. | N/A | 0.4 ml DMA 2.5, 5, 10 mg/kg/d | N/A |
|  |  | Wang et al, 2016 | DMA | National Institute for the Control of Pharmaceutical and Biological Products, Beijing, China | i.g. | N/A | 0.4 ml DMA | N/A |
|  |  | Zhao 2016 | SHI | Shanghai yuanye Bio-Technology Co., Ltd | i.p. | N/A | 0.1 ml SHI 5, 10 mg/kg/d | N/A |
|  |  | Yu et al, 2019 | SHI | Sigma-Aldrich, USA | i.g. | N/A | SHI 5, 10 mg/kg/d | N/A |
|  |  | Zhang et al, 2019 | SHI | N/A | i.g. | 20 mg/ml | 6.25, 12.5, 25 mg/kg/d | Shikonin of 98% purity, as verified by HPLC (Sigma, St. Louis, MO, USA), was dissolved in dimethyl sulfoxide (DMSO) at a stock concentration of 20 mg/ml. |
|  |  | Lan et al, 2020 | SO | Sigma-Aldrich; Merck KGaA | external use | 0.5766 mg/ml | N/A | Shikonin (Sigma‑Aldrich; Merck KGaA) was  dissolved in DMSO and diluted with MO (Fulinmen; COFCO Corporation) at 60˚C to produce the final concentration of SO. |
|  | *in vitro* | Xing, 2010 | SHI | WAKO Corporation | N/A | 30, 35, 40, 50, 65 μmol/L | N/A | N/A |
|  |  | Wang, 2011 | L-shikonin | China National Institute for the Control of Pharmaceutical and Biological Products | N/A | 10^-4^, 10^-5^, 10^-6^, 10^-7^ mol/L | N/A | Add 100 ul of absolute ethanol to dissolve every 3 mg L-shikonin, then add 10.3 ml of 5% DMEM to dilute to 10^-3^mol/L, sterilize with a 0.22 um pore size disposable filter, and further use 5% PBS DMEM for 10 diluted to 10^-4^, 10^-5^, 10^-6^, 10^N/A7^ mol/l. |
|  |  | Zhu, 2013 | SHI | Calibiochem, USA, product number 565850 | N/A | 10 mg/ml | N/A | 10 mg of comfrey was dissolved in 1 ml of DMSO, the storage concentration was 10 mg/ml, packaged separately, and stored in the dark at -20 °C for later use. |
|  |  | Xu et al, 2014 | SHI | N/A | N/A | 0-1 mmol/L | N/A | N/A |
|  |  | Xie et al, 2015 | SHI | Institute for Food and Drug Control | N/A | 1, 2 mg/L | N/A | Dissolve shikonin in DMSO below 8%. |
|  |  | Zhao et al, 2016 | L-shikonin | Beijing Institute of Drug Control | N/A | 10^-4^ mol/L | N/A | N/A |
|  |  | Liu et al, 2017 | SHI | Sigma, USA | N/A | 0, 1, 5, 10 μmol/L | N/A | N/A |
|  |  | Yu, 2019 | SHI | Sigma, USA | N/A | 0, 0.5, 1, 2, 4, 8, 10 mmol/L | N/A | Dissolve shikonin in DMSO to 20 mg/ml as a storage concentration and dilute to working concentration before use. |
|  |  | Lan et al, 2020 | SHI | Sigma-Aldrich; Merck KGaA | N/A | 0, 0.05, 0.25, 0.50, 1, 2.5, 5 mmol/L | N/A | N/A |
|  |  | Wang et al, 2014 | SHI | China National Institute for the Control of Pharmaceutical and Biological Products | N/A | 50, 100μmol/ml | N/A | N/A |
|  |  | Wang et al, 2016 | DMA | National Institute for the Control of Pharmaceutical and Biological Products, Beijing, China | N/A | 5, 10μg/ml | N/A | N/A |
|  |  | Qu, 2010 | SHI | WAKO Corporation | N/A | 0.2, 0.8, 2, 5, 7.5, 10, 12.5, 20, 20μg/ml | N/A | Dissolve 10 mg shikonin in 1 ml DMSO, fill to volume with double distilled water, the final concentration of DMSO in the cell culture medium is less than 0.5%. |
|  |  | Zhang, 2011 | SHI | WAKO Corporation | N/A | 2.5, 7, 12.5μg/ml | N/A | Shikonin is dissolved in DMSO, the final concentration of DMSO is less than 0.05%. |
|  |  | Wang, 2017 | DMA | State Food and Drug Administration | N/A | 12.5μg/ml | N/A | N/A |

**Abbreviations:** i.g., intragastric administration; i.p., intraperitoneal injection; N/A, Not applicable; HPLC, high performance liquid chromatography; SO, shikonin oil; MO, medium oil; PBS, phosphate-buffered saline; DMEM, dulbecco's modified eagle medium; SHI, shikonin; DMA, β,β-dimethylacryloyl alkannin.
