# Supplementary material for: Evidence and Potential Mechanism of Action of Lithospermum erythrorhizon and Its Active Components for Psoriasis
Source: Front Pharmacol. 2022 May 5;13:781850. doi: 10.3389/fphar.2022.781850 (PMC9128614; doi:10.3389/fphar.2022.781850)
Supplement: Supplementary file 6 [file Table3.docx]

| **Table S3. The characteristics of included preclinical studies in vivo** | | | | | | | |
| --- | --- | --- | --- | --- | --- | --- | --- |
| **Study(years)** | **Species (sex, n = experimental/ control group)** | **Weight(g)** | **Model (method)** | **Anesthetic** | **Method of administration** | | **Outcome index (time)** |
|  |  |  |  |  | **Experimental group** | **Control group** |  |
| Wang *et al*,  2015 | BALB/c mice （male, 18/18) | 18-20 | Psoriasis model(42mg/d 4% IMQ, 6d) | pentobarbital sodium, 80 mg/kg, i.p. | 0.4ml DMA 2.5, 5, 10 mg/kg/d, qd, i.g., apply 4% IMQ on the back 42mg/d, 6d | CON group: 0.4 mL NS, qd, i.g., apply Vaseline on the back, 6d IMQ group: apply 4% IMQ on the back 42mg/d, 6d MTX group: 0.4 mL MTX 1mg/kg/, qd, i.g., apply 4% IMQ on the back 42mg/d, 6d | 1.PASI 2.Epidermal thickness 3.IL-23 |
| Wang *et al*,  2016 | BALB/c mice （male, 6/18) | 18-20 | Psoriasis model(42mg/d 4% IMQ, 6d) | pentobarbital sodium, 80 mg/kg, i.p. | 0.4ml DMA, qd, i.g., apply 4% IMQ on the back 42mg/d, 6d | CON group: 0.4 mL NS, qd, i.g., apply Vaseline on the back, 6d IMQ group: 0.4 mL NS, qd, i.g., apply 4% IMQ on the back 42mg/d, 6d MTX group: 0.4 mL MTX 1mg/kg/, qd, i.g., apply 4% IMQ on the back 42mg/d, 6d | 1.CD11C+DC 2.TLR7 3.TLR8 4.MyD88 5.IRAKM |
| Zhao 2016 | BALB/c mice （female, 18/27) | N/A | Psoriasis model(42mg/d IMQ, 8d) | N/A | 0.1ml SHI 5, 10mg/kg/d, i.p., apply IMQ on the back 42mg/d | CON group：0.1ml NS, i.p.; apply Vaseline on the back 42mg/d IMQ group：0.1ml NS, i.p.; apply IMQ on the back 42mg/d MTX group：0.1ml MTX 1mg/kg/ｄ, i.p., apply IMQ on the back 42mg/d | 1.PASI 2.The HE dye 3.Epidermal thickness 4.IL-17 5.IL-17F 6.TNF-α 7.IL-6 8.IL-22 9.IL-23 10.CXCL1 11.CXCL5 12.MMP3 13.IL-17A |
| Yu *et al*,  2019 | BALB/c mice （male, 12/18) | N/A | Psoriasis model(62.5mg/d 5% IMQ, 10d) | N/A | SHI, 5, 10 mg/kg/d, i.g., apply 5% IMQ on the back 62.5mg/d, 10d | CON group: N/A  IMQ group: apply 5% IMQ on the back 62.5mg/d, 10d MTX group: MTX 0.5mg/kg/, qd, i.g., apply 5% IMQ on the back 62.5mg/d, 10d | 1.PASI 2.Epidermal  thickness 3.P-STAT3 4.STAT3 5.CEBPD |
| Zhang et al, 2019 | BALB/c mice （male, 18/12) | 17-20 | Psoriasis model(62.5mg/d 5% IMQ, 10d) | N/A | 6.25, 12.5, 25mg/kg/d SHI, i.g., apply IMQ on the back 62.5mg/d, 10d | CON group: 5% DMSO in oil, i.g., apply topical white petrolatum on the back, 10d IMQ group: 5% DMSO in oil, i.g., apply IMQ on the back 62.5mg/d, 10d MTX group: 0.5 mg/kg/d MTX, i.g., 10d, apply IMQ on the back 62.5mg/d, 10d | 1.PASI 2.The HE dye  3.Spleen Index 4.CD25Foxp3 6.IL-10 7.TGF-β-1 8.Foxp3 9.IL-6 10.IL-17A |
| Lan *et al*,  2020 | BALB/c mice （male, 5/15) | 20-25 | Psoriasis model(50mg/d 5% IMQ, 8d) | N/A | 0.5ml SO,external use, apply 5% IMQ on the back 50mg/d, 8d | CON group: N/A MO group: 0.5ml MO, 8d, apply 5% IMQ on the back 50mg/d, 8d | 1.PASI 2.CEBPD 3.K17 |
| **Abbrevations:** qd, once a day; NS, normal saline; IMQ, Imiquimod cream; i.g., intragastric administration; i.p., intraperitoneal injection; N/A, Not applicable; DMA, β, β-dimethylacryloyl alkannin; SO, shikonin oil; MO, medium oil; PBS, phosphate-buffered saline; SHI, shikonin; CON, control; IL, interleukin; CEBPD, CCAAT/enhancer-binding proteinδ; K17, keratin 17. | | | | | | | |
