# Supplementary material for: Evidence and Potential Mechanism of Action of Lithospermum erythrorhizon and Its Active Components for Psoriasis
Source: Front Pharmacol. 2022 May 5;13:781850. doi: 10.3389/fphar.2022.781850 (PMC9128614; doi:10.3389/fphar.2022.781850)
Supplement: Supplementary file 7 [file Table4.docx]

| **Table S4. The characteristics of preclinical studies in vitro** | | | | |  |
| --- | --- | --- | --- | --- | --- |
| **Author, year** | **Cell Type** | **Cell origin (species)** | **Cell Tracking Method** | **Outcomes** | **Pathway** |
| Xing, 2010 | HaCaT | Human normal skin immortalized KC | ELISA (IL-17, IL-6, IL-23) | SHI inhibited the expression of VEGF, IL-6 and IL-23 in HaCaT cells stimulated by IL-17, and affected the formation and activation of Th17 cells | N/A |
| Wang, 2011 | HaCaT | Human normal skin immortalized KC | FCM (cell cycle of HaCaT) | 1. shikonin inhibits the proliferation of HaCaT cells induced by KGF by blocking the transformation of HaCaT cells to the S phase and G2M phase and making the cell growth stop at the G0G1 phase | N/A |
| Zhu, 2013 | HaCaT | Human normal skin immortalized KC | PCR (S100A7, S100A8, S100A9)  WB (Phosphorylation of STAT3Try705 tyrosine , STAT3Ser727 serine, Erk1/2 and P38)  WB (Gab1, Gab2) | SHI inhibited IL-22-induced HaCaT cell proliferation and migration, and down-regulated IL-22-induced S100A7 and S100A8 mRNA expression.  SHI may interfere with the biological effects of IL-22 directed HaCaT cells by inhibiting the activation of Gab1 and Gab2, and further inhibiting the activation of the Erk1/2 /MAPK pathway. | Erk1/2 /MAPK pathway |
| Xu et al, 2014 | HUVECS, HaCaT | human umbilical cord blood, Human normal skin immortalized KC | WB (IL-17R, P-JAK2, P-STAT3, SOCS1) ELISA (VEGF) | SHI can inhibit the expression of VEGF in HaCaT cells induced by IL-17 and the secretion of VEGF in HaCaT cells, inhibit the expression of IL-17R, PJAK2 and PSTAT3 induced by IL-17, and up-regulate the expression of SOCS1 in HaCaT cells | JAK2/STAT3 pathway |
| Xie et al, 2015 | HaCaT | Human normal skin immortalized KC | ELISA (IL-23) PCR (CXCL1, CXCL2, CCL20, DEFB4) | SHI can reduce the secretion of IL-23 and inhibit CXCL1, CXCL2, CCL20, DEFB4 | N/A |
| Zhao et al, 2016 | HaCaT | Human normal skin immortalized KC | PCR (HBD-2) ELISA (HBD-2) WB (JNK) | 1. shikonin inhibited the activation of JNK2/MAPK signaling pathway and down-regulated the expression of HBD-2 | JNK2/MAPK pathway |
| Liu et al, 2017 | HaCaT | Human normal skin immortalized KC | WB (PCNA) | SHI inhibited the up-regulation of PCNA protein expression, and it may inhibit the pro-proliferation effect of EGF on HaCaT cells by inhibiting the expression of miR-21 | N/A |
| Yu, 2019 | HaCaT | Human normal skin immortalized KC | PCR (STAT3, CEPBD) WB (P-STAT3, STAT3, CEPBD, cleaved Caspase-9, CyclinE) FCM (Apoptosis and cell cycle of HaCat) | SHI inhibited the proliferation and promotes the apoptosis of HaCaT cells, block HaCaT cells in G0/G1 phase and reduce the cell distribution in G2/M phase, inhibited the proliferation of HaCaT and promote apoptosis by inhibiting the JAK/STAT3 pathway and up-regulating the expression of CEBPD | JAK/STAT3 pathway |
| Lan et al, 2020 | HaCaT | Human normal skin immortalized KC | WB (CEBPD, K17) | SHI reverses the down-regulation of IL-17-mediated tumor suppressor CEBPD in HaCaT cells. | N/A |
| Wang et al, 2014 | DC | Human peripheral blood monocytes | FCM (CD80, CD83, CD86) ELISA (IL-23) | SHI inhibited the expression of dendritic surface molecules, such as CD86, CD80, and CD83. SHI can reduce the secretion of IL-23. | N/A |
| Wang et al, 2016 | DC | Mouse bone marrow cells | FCM (I-E/I-A, CD80, CD86)  PCR (IL-1β, IL-23)  CBA (IL-1β, IL-23)  WB (TLR7, TLR8, MyD88, IRAKM) | DMA decreased I-E/I-A, CD80 and CD86 on the cell surface of activated DCS.  DMA inhibited the secretion of IL-23 and IL-1β after R848 stimulation.  DMA inhibited the expression of TLR7, MyD88 and IRAKM after R848 stimulation. | N/A |
| Qu, 2010 | PBMCs | Human peripheral blood | ELISA (IL-23, IL-6, IL-17) | SHI inhibits IL-6 and IL-17 production in PBMCs induced by IL-23 in patients with psoriasis | N/A |
| Zhang, 2011 | PBMCs | Human peripheral blood | ELISA (IL-4, IFN-γ) | SHI can inhibit the secretion of Th1 cytokines and increase the secretion of Th2 cytokines | N/A |
| Wang, 2017 | PBMCs | Human peripheral blood | FCM (CD80, CD83, CD86) PCR (IL-6, IL-12p40, IL-1 β), TNF- α, IL-23, IL-10, IL-6, IL-10, IL-1 β, IL-23, TNF- α) | DMA inhibited the activation and function of murine bone-derived dendritic cells by inhibiting the TLR7/8 pathway and the downstream NF-kB | TLR7/8 pathway |
| Wu et al, 2003 | colo-16 | Human skin squamous cells | FCM (hypodiploid of colo-16) | SHI can induce apoptosis of epidermal Colo-16 cells | N/A |
| Sun et al, 2014 | colo-16 | Human skin squamous cells | FCM (hypodiploid of colo-16) | SHI can induce apoptosis of epidermal Colo-16 cells | N/A |
| Liu et al, 2018 | Jurkat E6-1 | Human T lymphocytes | FCM (CD69) ELISA (IL-2, IFN-γ, TNF-α)  WB (NF-AT, c-Jun, NF-κB) | SHI inhibited cell proliferation, CD69 expression and Th1 cytokine secretion, the expression of NF-AT, c-Jun and NF-κB | N/A |

**Abbrevations**: KC, keratinocyte cell; DC, Dendritic Cells; PBMCs, Peripheral blood mononuclear cell; HUVECS, Human Umbilical Vein Endothelial Cells; SHI, Shikonin; DMA, β,β-dimethylacryloyl alkann. ELISA,enzyme-linked immuno sorbent assay; FCM, Flow Cytometry; WB, Western Bolt; PCR, Polymerase Chain Reaction; IL, interleukin; SOCS, Suppressor of cytokine signaling; VEGF, vascular endothelial growth factor; PCNA, Proliferating Cell Nuclear Antigen; K17, Keratin 17; TLR, Toll like receptor; MyD88, Myeloiddifferentiationfactor88; HBD-2, human β-defensin-2; IRAKM, interleukin-1 receptor-associated kinase M.
